# High-throughput screening for optimizing adoptive T cell therapies

**DOI:** 10.1186/s40164-024-00580-w

**Published:** 2024-11-13

**Authors:** Yuchen Zhang, Qinglong Xu, Zhifei Gao, Honghao Zhang, Xiaoling Xie, Meifang Li

**Affiliations:** Department of Hematology, Zhujiang Hospital, Southern Medical University, Guangzhou, 510282 People’s Republic of China

**Keywords:** High-throughput screening, Adoptive T cell therapies, Genetic screening, Non-genetic screening, CRISPR screening, CAR-T cells, TCR-T cells

## Abstract

Adoptive T cell therapy is a pivotal strategy in cancer immunotherapy, demonstrating potent clinical efficacy. However, its limited durability often results in primary resistance. High-throughput screening technologies, which include both genetic and non-genetic approaches, facilitate the optimization of adoptive T cell therapies by enabling the selection of biologically significant targets or substances from extensive libraries. In this review, we examine advancements in high-throughput screening technologies and their applications in adoptive T cell therapies. We highlight the use of genetic screening for T cells, tumor cells, and other promising combination strategies, and elucidate the role of non-genetic screening in identifying small molecules and targeted delivery systems relevant to adoptive T cell therapies, providing guidance for future research and clinical applications.

## Introduction

Cancer immunotherapy has advanced rapidly in recent years, with the interactions between immune cells and tumor cells playing a crucial role in its effectiveness [1]. Adoptive cell transfer (ACT) involves collecting immune cells from either the patient or a donor, culturing or modifying them ex vivo, and then reintroducing them into the patient to enhance their ability to eliminate tumor cells. Particularly noteworthy in this area are T cell-based ACTs, such as Tumor Infiltrating Lymphocytes (TILs), engineered T Cell Receptor (TCR) T cell therapy, and Chimeric Antigen Receptor (CAR) T cell therapy [2]. These approaches have shown significant clinical effectiveness in treating hematologic malignancies [3]. However, adoptive T cell therapy is not always effective against certain hematologic malignancies and most solid tumors, often leading to the development of primary resistance. Possible mechanisms for this resistance include impaired T cell proliferation and differentiation, reduced cytotoxicity, the emergence of exhausted T cell phenotypes [4, 5], as well as factors such as downregulation of antigens and internal inhibitory pathways in tumor cells [6]. Consequently, understanding the positive and negative signals that govern the interaction between T cells and tumor cells, and thereby enhancing T cell effector functions, is a critical research focus in cancer immunotherapy.

High-throughput screening technology offers an unbiased platform for the large-scale identification of optimal genetic targets and other substances, encompassing both genetic and non-genetic screening approaches. This technology significantly enhances the efficiency and scope of research. Genetic screening involves designing and introducing extensive gene editing libraries into cell pools to identify biologically significant sites for further validation through functional experiments. This approach overcomes the limitations of editing single gene sites and substantially advances our understanding of cancer genomics [7]. The protein engineering of conventional zinc finger nucleases and transcription activator-like effector nucleases requires a complex design and construction process to determine the amino acid substitutions needed for selective binding to target genomic sequences. In contrast, the CRISPR/Cas9 (Clustered Regularly Interspaced Short Palindromic Repeats/CRISPR-associated 9) system, known for its flexibility and editing efficiency, has emerged as the leading high-throughput genetic screening method currently in use [8]. As CRISPR technology continues to evolve, new methods such as base editors (BEs) and epigenetic editors—techniques that do not rely on double-strand breaks—offer viable solutions to technological challenges. These advancements have found extensive applications in adoptive T cell therapy and related strategies [9]. By integrating genetic and non-genetic screening approaches, researchers can deepen their understanding of drug combination therapies and targeted delivery systems. This review highlights the latest technologies in high-throughput genetic and non-genetic screening and discusses recent developments in applying these technologies to T cells, tumor cells under T cell pressure, and other promising combination strategies. It aims to lay the groundwork for future clinical advancements.

## Current adoptive T cell therapies’ applications and limitations

Adoptive T cell therapy primarily includes three modalities: TILs, TCR-T, and CAR-T therapies. TIL therapy involves the ex vivo expansion of tumor-reactive T cells isolated after tumor resection, enhancing T cell infiltration and cytotoxicity against solid tumors with a generally favorable safety profile. However, its highly personalized nature results in elevated production costs, and clinical studies have largely focused on melanoma, where objective response rates are generally lower compared to CAR-T and TCR-T therapies [10]. A clinical trial (NCT03215810) involving non-small cell lung cancer patients demonstrated promising results: among 13 evaluable patients, 3 exhibited confirmed responses, and 11 experienced tumor burden reduction, with a median best change of 35%, indicating notable clinical efficacy [11]. To mitigate the limitations imposed by the external microenvironment and internal inhibitory signals, genetic ablation of RASA2 in T cells has been shown to enhance their antigen sensitivity and persistence [12].

CAR-T therapy has shown significant potential in treating various hematologic malignancies and select solid tumors, particularly in B-cell malignancies. To date, six CAR-T therapeutic products have been approved by the U.S. Food and Drug Administration (FDA), demonstrating remarkable clinical efficacy and safety profiles [13]. However, CAR-T therapy also faces challenges such as severe cytokine release syndrome and neurotoxicity, which require ongoing management. TCR-T therapy, on the other hand, can recognize both cell surface antigens and intracellular tumor antigens presented by peptide-major histocompatibility complexes, making it particularly suitable for targeting solid tumors. The FDA has approved Kimmtrak for treating patients with metastatic uveal melanoma, highlighting the clinical potential of this approach [14]. Nevertheless, TCR-T therapy faces challenges such as potential off-target effects and the need for precise antigen targeting. Gene editing technologies have become crucial in addressing the limitations of both CAR-T and TCR-T therapies. For example, CRISPR/Cas9-mediated knockout of programmed cell death 1 (PD-1) in mouse models is a prevalent method to enhance anti-tumor responses and reduce CAR-T cell exhaustion. Additionally, CAR-T cells with the CAR construct integrated into the PD-1 locus have demonstrated promising efficacy in both preclinical and clinical studies [15]. In TCR-T cell therapy, CRISPR/Cas9 technology has been used to simultaneously ablate endogenous TCR genes while introducing neoantigen-specific TCR genes. Clinical trials (NCT03970382) have shown that some patients exhibit disease remission with this approach [16]. Overall, while current adoptive T cell therapies offer substantial promise, they also face specific challenges and limitations. Advances in gene editing and continued research are essential for optimizing these therapies and addressing their current constraints.

Despite the impressive preclinical anti-tumor efficacy and clinical successes of adoptive T cell therapies, several significant challenges persist. The most prevalent issues include the development of resistance or post-treatment relapse, which limits therapeutic efficacy. These outcomes are primarily due to the functional decline of persistently infiltrating T cells and mechanisms of antigen escape or downregulation [17]. Antigen-positive relapse or resistance is often associated with T cell exhaustion, suboptimal CAR potency, and reduced tumor responsiveness to CAR-T cells. For example, studies have shown that CARs with 4-1BB costimulatory domains exhibit enhanced persistence compared to those with CD28 costimulatory domains [18]. Conversely, antigen-negative relapse or recurrence may be caused by genetic mutations or trogocytosis-mediated antigen loss [19]. To address antigen-positive relapse or resistance, strategies such as T cell functional screening, CAR structural optimization, and intratumoral functional assessment are promising. Additionally, investigating antigen presentation mechanisms and other relevant intracellular pathways may offer strategies to mitigate antigen-negative relapse or resistance. Furthermore, adverse events such as cytokine release syndrome, immune effector cell-associated neurotoxicity syndrome, and off-target tumor toxicity raise safety concerns regarding cellular therapies [20]. In conclusion, employing high-throughput screening methodologies to identify viable gene targets for promotion or inhibition, as well as potential drugs or delivery vectors, is a crucial direction for advancing CAR-T cell therapy. These efforts aim to enhance the efficacy and safety of CAR-T treatments, improve their accessibility, and ultimately broaden the therapeutic landscape of this promising approach.

## Advanced technologies of high-throughput screening

### Genetic screening

#### Mechanism and features

Genetic screening, an effective tool for identifying unknown targets and mechanisms, is distinguished by its high throughput and efficiency, encompassing small interfering RNA (siRNA), short hairpin RNA (shRNA), and, most prominently, CRISPR screening. siRNAs are synthetically produced short RNA fragments typically employed in independent phenotypic screenings using 96-well or 384-well culture plates. Alternatively, commercially available siRNA libraries, where each siRNA possesses a unique barcode, can be utilized. The efficacy of specific siRNAs is determined by quantifying their respective barcodes. However, this approach is limited in scope, operationally complex, and cost-prohibitive for large-scale experiments [21]. shRNAs offer a more streamlined and efficient approach through the construction of mixed libraries using viral vectors, with data acquisition via deep sequencing. However, RNAi techniques, including shRNAs, face limitations in high-throughput applications due to incomplete gene expression suppression, resulting in unstable phenotypes. Additionally, high off-target effects compromise the accuracy of these methods, rendering their practical application suboptimal [22]. In contrast, the currently predominant CRISPR screening methodology offers versatile applications including knockout (KO), knock-in (KI), activation, and inhibition, providing diverse and robust modes of action. Moreover, the design and construction of single guide RNA (sgRNA) libraries for CRISPR screening are relatively straightforward. Furthermore, CRISPR screening extends beyond targeting gene coding regions, enabling the interrogation of non-coding sequences such as promoters, enhancers, and long non-coding RNAs (lncRNAs), thereby elucidating their roles in gene expression regulation [23]. Additionally, the development of multiplex CRISPR libraries utilizing 4gRNA-combo (combinations of four gRNAs) enables simultaneous targeting of multiple gene pairs within a single cell, thereby enhancing the capacity to explore combinatorial effects of gene interactions [24]. However, the accessibility of CRISPR screening applications remains limited, with constraints on delivery and expression in certain cell types or organisms. For instance, large-scale expression in primary tumor cells and primary T cells continues to pose significant challenges, thereby restricting its application in adoptive T cell therapies for cancer treatment [25]. A comparative analysis of these three genetic screening methodologies is presented in Table 1.

**Table 1:** Comparisons of different screening methods

Screening methods	Advantages	Disadvantages	Research purpose
siRNA screening	1. Easy to construct with established technology 2. Rapid onset of action	1. Limited to independent phenotypic screening on culture plates or the use of commercial libraries, resulting in a narrow scope and high costs 2. Lower stability and higher off-target effects 3. Limited to targeting mRNA molecules 4. The interference effects of different genes vary across different cell types	1. Ideal for short-term gene silencing experiments 2. Quick validation of gene function
shRNA screening	1. Suitable for mixed library screening 2. Enhanced stability 3. Extended duration of action	1. Limited to gene silencing; cannot fully suppress gene expression to achieve a stable phenotype during high-throughput screening 2. Some off-target effects still exist 3. Prolonged expression may trigger non-specific immunity, resulting in cytotoxicity 4. Limited to targeting mRNA molecules 5. The interference effects of different genes vary across different cell types	1. Ideal for long-term gene silencing experiments 2. Enables controlled or reversible silencing experiments of gene expression
CRISPR screening	1. High gene editing efficiency and low off-target effects 2. Cost-effective and suitable for large-scale gene screening 3. Supports multiple editing forms, including KO, KI, activation, and inhibition 4. Capable of targeting non-coding sequences 5. Possesses potential for multiplex gene editing	1. CRISPR delivery and expression are restricted in certain cells or organisms 2. Requires a high level of technical skill 3. Unable to dynamically regulate gene expression 4. Chromosomal toxicity may arise following gene editing 5. Off-target effects continue to be a concern	1. Applications for precise and permanent gene alteration 2. Suitable for screening non-coding sequences 3. Multiplex gene pair screening, expanding the ability to explore combinations of gene interactions and behaviors

Libraries for whole-genome or large-scale functionally relevant gene edits are created and transduced into target cell pools, notably T cells and tumor cells. Screening conditions are applied, such as co-culturing tumor cells with T cells, continuous antibody or therapeutic drug stimulation, or in vivo screening in immune-competent or immune-deficient NSG mice, with blank or conditional controls, or occasionally using survival pressure for comparison. In the event of altered phenotypes due to gene editing, cells can be categorized using Fluorescence-Activated Cell Sorting (FACS) based on varying surface markers or cytokines produced. Following that, high-throughput sequencing technology counts sgRNAs or other gene editing components in each group, reads barcodes after Polymerase Chain Reaction (PCR) amplification, and analyzes the depletion or enrichment of sgRNAs in gene-edited cells. This process helps uncover genes linked to T cell proliferation, activation, differentiation, and cytotoxicity in tumor immunity, as well as mechanisms behind tumor resistance to adoptive T cell therapy [7].

Genetic screening is categorized into loss-of-function (LOF) and gain-of-function (GOF) screening. Traditional siRNA, shRNA, and CRISPR/Cas9 systems typically silence or knock out genes within cells, aiming to identify genes in tumor cells sensitive to resistance or negative regulators of anti-tumor functions in other immune cells. Although LOF screening enhances cell therapy, the translational potential of knocking in large gene cassettes to rewrite cellular programming is more significant. GOF screening chiefly utilizes CRISPR-mediated gene activation (CRISPRa) methods for temporary gene transcription activation. Nevertheless, the large size and immunogenicity of many Cas9 transcription activator fusion proteins restrict their use in screenings, necessitating a robust CRISPR KI system for GOF screening. Roth et al. developed a parallel targeting library of KI protein constructs at the same site, utilizing barcodes and tracking of large non-viral DNA templates. This identified genes enhancing T cell adaptability and allowed for efficient and precise GOF pooled screening [26]. Employing a lentiviral library with barcoded human open reading frames (ORFs) enables genome-scale GOF screening in primary human CD4^+^ and CD8^+^ T cells. A novel single-cell sequencing method, integrated with direct ORF capture, has been developed, broadening the scale and scope of GOF applications [27]. The ongoing advancements in genetic screening technology have been instrumental in laying the groundwork for the evolution of tumor immunotherapy techniques.

**Fig. 1:**
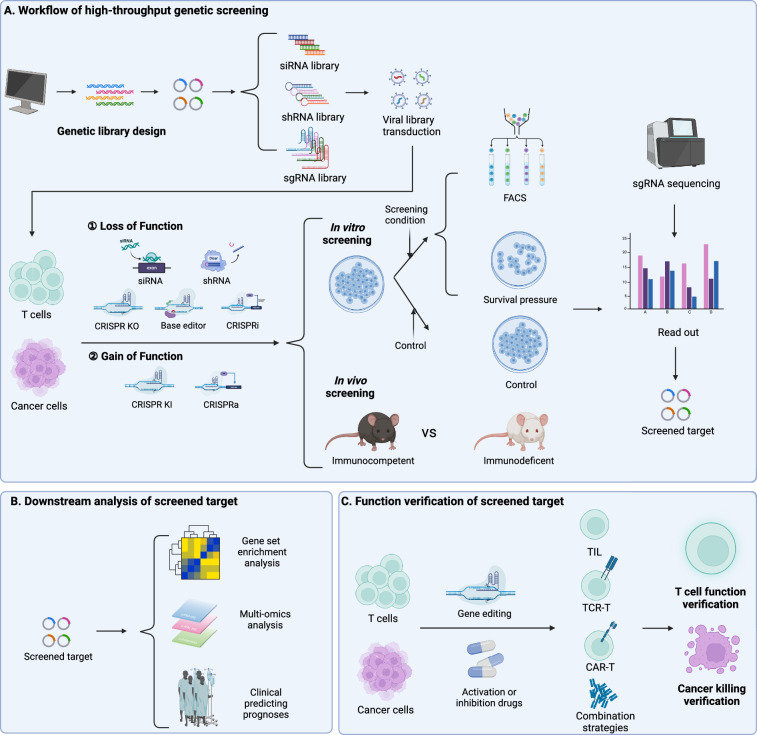
Advanced technologies of high-throughput genetic screening. **A** Libraries of siRNA, shRNA, and sgRNA were designed and constructed for delivery into tumor cells or T cells using viral vectors. The screening was categorized into LOF and GOF approaches, utilizing siRNA, shRNA, CRISPR KO, base editing, or CRISPR interference (CRISPRi) for the former, and CRISPR KI and CRISPRa for the latter. In vitro screening involved comparing experimental groups under specific conditions with control groups or sorting based on surface markers or other phenotypes using FACS. For in vivo screening, comparisons were made between immunocompetent and immunodeficient groups, with the identification of screened targets achieved through sgRNA sequencing. **B** Downstream analysis of the screened targets primarily encompassed GSEA, multi-omics analysis, and clinical prognosis prediction. **C** In T cells and tumor cells, genetic editing on screened targets or the application of activating or inhibitory drugs aims to enhance adoptive T cell therapy and other promising combination strategies, affirming T cell functionality and tumoricidal efficacy

Downstream analysis, a vital step post-target identification, typically uses gene set enrichment analysis (GSEA) to identify functional pathways in T cell-mediated tumor cell cytotoxicity and validate key phenotypes of related functions. For instance, employing CRISPR/Cas9 for cellular engineering or small molecule drugs to modulate targets can confirm their effects on positive aspects like T cell activation, differentiation, and tumor cytotoxicity, as well as on negative states like exhaustion. Additionally, these findings are implemented in adoptive T cell therapies or other combination strategies, aiming to enhance the efficacy of adoptive T cell therapy. The advancement of high-throughput sequencing and multi-omics technologies has propelled the development of screening precision and research depth [28]. Most CRISPR screening methods depend on bulk sgRNA readouts from selected cell phenotypes, thus often neglecting the heterogeneity among cells. Contrastingly, single-cell CRISPR (scCRISPR) screening methods combine gene perturbations with single-cell RNA sequencing (scRNA-seq), categorizing into four main types based on different single-cell technologies: scCRISPR with RNA-seq, scCRISPR with Assay for Targeting Accessible-Chromatin with high-throughput sequencing, scCRISPR with proteomics, and imaging-based scCRISPR [29]. These methods enable transcriptomic analysis of individual genetic perturbations within complex cell pools, offering detailed molecular insights into targeted disruptions at the single-cell level, and have achieved notable progress in T cell research [25, 30–34] (Fig. 1).

Moreover, enhancing the sensitivity and efficiency of high-throughput screening technologies while minimizing potential biases remains a critical technical challenge. The large-scale nature of these technologies, combined with their high result specificity and diverse screening targets and applications, means that even minor sample variations can significantly influence result analysis. Consequently, these technologies face challenges such as reduced measurability due to high interference characteristics and limited reproducibility stemming from false-positive signals. The velocity and volume of data generation in high-throughput methodologies significantly surpass those of traditional experimental techniques. Therefore, standardization and reproducibility are paramount for ensuring data reliability. As screening throughput expands, maintaining sample integrity and minimizing interference from extraneous factors require substantial technical discourse.

#### Emerging CRISPR screening tools

Traditional CRISPR/Cas9-mediated genome editing induces targeted DNA double-strand breaks (DSBs), resulting in numerous indel edits and genetic instability. Deactivated Cas9 (dCas9) or single-strand cutting nickase Cas9 (nCas9) can prevent DSBs, thus minimizing genetic mutation risks. DNA BEs are precise technologies for substituting single nucleotides, by fusing dCas9 or nCas9 with deaminases targeting single-strand DNA. Anchored by sgRNA to target sites, they induce GC-to-TA or AT-to-GC mutations for accurate nucleotide replacement in genes [35]. CRISPR screening uncovers genes impacting immune cell functions, but different alleles within the same gene can influence protein structure and function differently. Thus, base editing screening provides high-resolution analysis of individual genes' key nucleotides, facilitating precise modulation of protein function [36–38]. Additionally, CRISPRi incorporates the Kruppel-associated box transcription repression domain into dCas9, directing the complex to the transcription start site (TSS) via sgRNA, thereby reducing the expression of target genes. Conversely, CRISPRa employs transcriptional activators like VP64 at the TSS, with dCas9 engineered to fuse with various transcriptional activation domains such as RTA, VP64, HSF1, and p65. This fusion strategy enables the upregulation of specific genes across different cell types, selectively activating endogenous gene transcription mechanisms and enhancing target region expression [39]. Moreover, the fusion of dCas9 with epigenetic modifiers like DNA methyltransferases and ten-eleven translocation enzymes facilitates the development of epigenetic editing systems. This non-DNA-cutting method is reversible and avoids permanent genomic alterations. Unlike traditional gene knockout techniques, it minimizes severe extracellular effects and intrinsic phenotypic ambiguity. This approach can also identify genes that may be inactive under test conditions but influence phenotypic expression. Combining CRISPRa and CRISPRi screening can complement each other to reveal both positive and negative regulatory factors within cells [25, 40, 41].

Analogous to Cas9, Cas12a induces DSBs for gene editing but exhibits a more relaxed binding affinity to the target genome, demonstrating enhanced reversibility in enzyme-target sequence interactions. Cas12a requires only CRISPR RNA (crRNA) for its expression, eliminating the need for tracrRNA and reducing system complexity. This simplification results in lower cloning costs and mitigates delivery challenges. Consequently, CRISPR-Cas12a has emerged as a potent and promising gene editing tool. Additionally, the recently discovered Cas13d demonstrates precise message RNA (mRNA) targeting and degradation capabilities. Its high efficiency in gene expression interference positions Cas13d as a potential successor to traditional RNA interference technologies. Through the construction of multi-crRNA expression arrays, Cas13d exhibits the capacity for multi-gene expression regulation, offering an expansive editing scope. As a compact RNA editing system, Cas13d offers significant advantages in vector delivery [42].

### Non-genetic screening

In adoptive T cell therapy, the integration of genetic and non-genetic screening underpins combination therapy and affinity research. Large-scale drug sensitivity screening reveals the mechanisms of drug therapy combined with tumor immunotherapy at the genomic level. Tumor cells labeled with luciferase (Luc), T cells, and a drug library are co-cultured in plates like 96-well or 384-well. By measuring Luc activity, drugs that enhance adoptive T cell therapy and improve tumor cell killing are selected. CRISPR screening then identifies specific mechanisms, uncovering the immunomodulatory traits of cancer drugs.

Additionally, non-genetic screening of chemical non-viral delivery systems is performed using synthetic chemical combinatorial libraries. Initially, physicochemical properties are characterized and screened in vitro using techniques such as dynamic light scattering, transmission electron microscopy, and chromatography. These methods assess properties like lipid nanoparticle (LNP) size, zeta potential, and pKa. Following this, a combination of in vitro and in vivo experiments monitors protein expression in external cells and the system’s delivery targeting to internal organs and cells. This approach helps identify the optimal structure of innovative non-viral delivery systems, enhancing targeting capabilities and providing a platform for thorough characterization and screening of the delivery system’s specificity and efficiency.

## Genetic screening applied for T cells

### T cell screening with cytotoxic functions

**Fig. 2:**
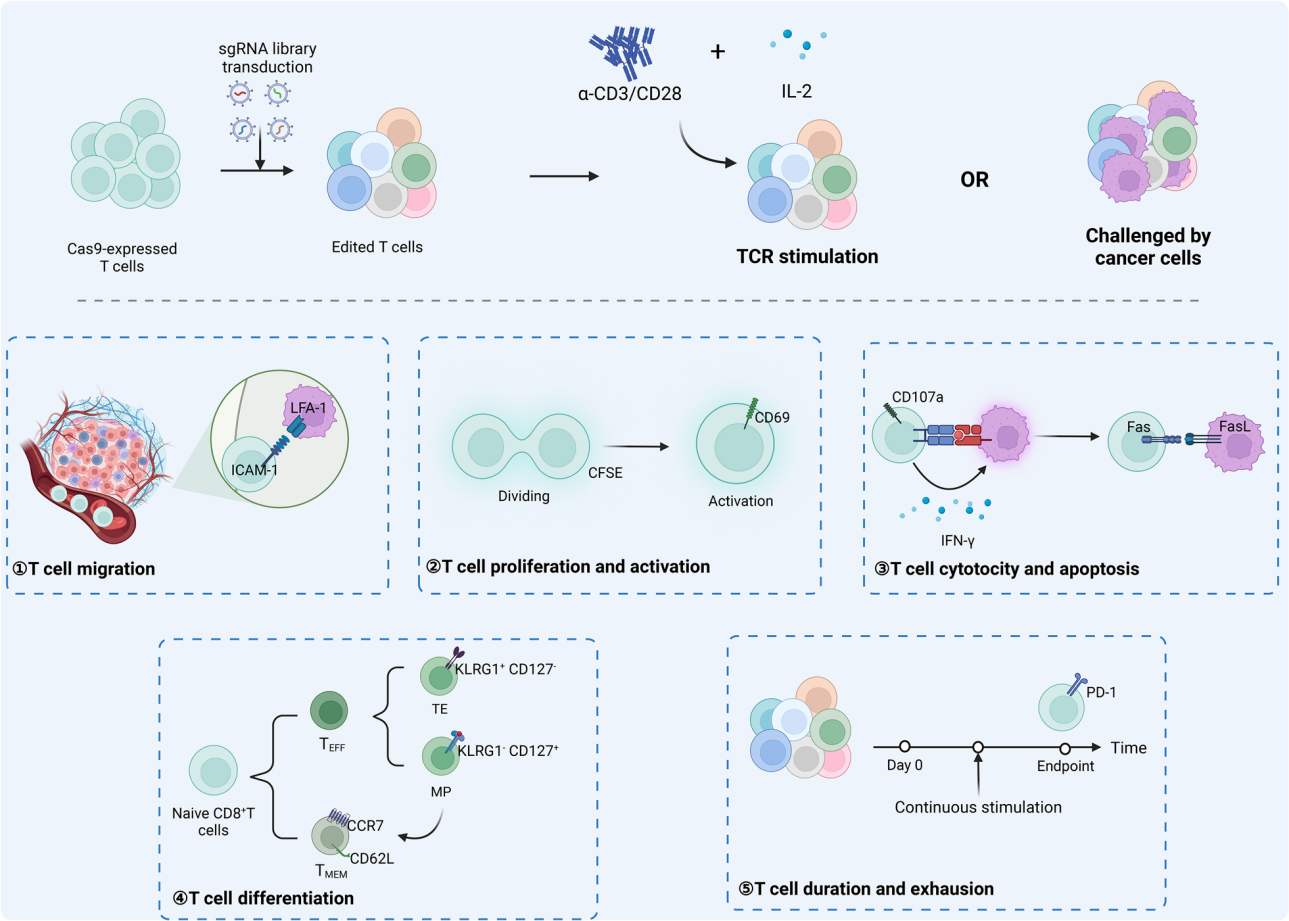
T cell screening with cytotoxic functions. After introducing the viral library, T cells with gene edits are obtained. These edited T cells can be stimulated via TCR activation using α-CD3/CD28 and IL-2 or co-cultured directly with tumor cells under specific conditions. For evaluating T cell homing, screening for ICAM-1 or analyzing in vivo distribution is performed. Cell proliferation and activation are assessed using the CFSE method and CD69 expression, while CD107a and IFN-γ serve as classic phenotypic markers of T cell cytotoxicity. The Fas pathway also plays a crucial role in cellular apoptosis. CD8^+^ T cells T_EFF_ and T_MEM_, with CCR7 and CD62L aiding in identifying regulators of T_MEM_ differentiation and maintenance. T_EFF_ cells can be further divided into short-lived effector cells (TE; KLRG1^+^ CD127^−^) and memory precursor cells (MP; KLRG1^−^ CD127^+^), with MPs evolving into T_MEM_ to provide durable protective tumor immunity. Continuous in vitro assays and temporal comparisons help identify enduring targets in T cell therapy, particularly focusing on exhausted cells that express inhibitory factors like PD-1

Cytotoxic CD8^+^ T cells are crucial in tumor immunity, integrating both positive and negative signals to facilitate functions such as proliferation, activation, infiltration, and targeted cytotoxicity [43]. High-throughput genetic screening of T cells is conducted through TCR stimulation with anti-CD3/CD28 and IL-2, co-culturing with tumor cells (in vitro), or implantation in tumor-bearing mice (in vivo). Phenotypes are sorted using FACS, or cell groups under various screening conditions are sequenced and compared to identify genes regulating specific functional phenotypes. Single gene editing has been employed to enhance TIL and CAR-T therapies, demonstrating increased success rates in treating tumors with adoptive T cell therapy, as validated in both in vitro and in vivo models (Table 2 and Fig. 2).

**Table 2:** Representative Studies of Genetic Screening for T cell screening with cytotoxic functions

Screening methods	Screening cells	Screening model	Library design	Screening strategies	Comparison and readout	Screened targets	Function of Screened target	Refs.
CRISPR KO	OT-I T cells	in vivo	Activates differentially expressed gene library between CD8 T cells and naïve CD8 T cells	T cells were injected into B16-OVA mice, and the distribution of T cells was observed	Compare the lung/liver group, the stay in the circulation group, and the untreated group	St3gal1	St3gal1 regulates LFA-1 mediated CAR-T cell migration	[45]
CRISPR KO	OT-I T cells	in vitro	Gene library of PI3K-related pathways	TCR and scICAM were stimulated, FACS sorts ICAM expression	Compare ICAM^pos^ group and ICAM^neg^ group	RASA3	RASA3 is a negative regulator of LFA-1-mediated adhesion in T cells	[46]
CRISPR KO	OT-I T cells	in vitro	Genome-wide library	TCR stimulation, FACS sorts CFSE expression	Compare dividing T cells and non-dividing T cells	RASA2	RASA2 ablation in T cells boosts antigen sensitivity and long-term function	[12]
CRISPRi	Jurkat and primary human T cells	in vitro	Genome-wide library	TCR stimulation, FACS sorts GFP expression	Compare GFP^high^ group and GFP^low^ group	NAMPT	NAMPT is required for T cell activation	[47]
CRISPRa	Primary human CD4^+^ and CD8^+^ T cells	in vitro	Human ORF Library	Pre-stimulation, FACS sorts CFSE expression	Compare dividing T cells and non-dividing T cells	LTBR	LTBR increase T-cell effector functions, and resistance to exhaustion in chronic stimulation	[27]
CRISPR KO	OT-I T cells	in vitro	Kinase-related gene library	TCR stimulation, FACS sorting cell proliferation, CD62L, ROS, γH2AX expression	Compare the four phenotypic high-expression and low-expression groups	P38	p38 kinase is a central regulator of all four phenotypes, including cell expansion, differentiation, oxidative stress, and genomic stress	[48]
CRISPRi	Jurkat t cells	in vitro	Genome-wide libraries	TCR stimulation, FACS sorting CD69	Compare CD69^high^ group and CD69^low^group	FAM49B	FAM49B is a key regulator of actin dynamics and T cell activation	[49]
Base editing	Primary human CD8^+^ T cells	in vitro	T cell activity-related gene libraries	TCR stimulation, FACS sorting TNF, IFN-γ, PD-1, CD25 expression	Compare the production of TNF and IFN-γ, the high and low surface expressions of CD25 and PD-1	PIK3CD, VAV1, LCP2, PLCG1,DGKZ	Reduce T cell cytotoxic function	[36]
CRISPR KO	OT-I T cells	in vivo in vitro	Genome-wide libraries	T cells are injected into Rag1- mice bearing E0771-mCh-OVA to observe T cell killing T cells were co-cultured with SIINFEKL-pulsed E0771 cells, and FACS sorted CD107a expression	Direct readout to survival T cells in vivo Compare CD107a^high^ group and CD107a^low^ group	DHX37	DHX37 modulates NF-kB to suppress effector functions, cytokine production, and T cell activation	[50]
CRISPRa	OT-I T cells	in vitro	Genome-wide libraries	T cells were co-cultured with SIINFEKL-pulsed E0771 cells, and FACS sorted CD107a expression	Compare CD107a^high^ group and CD107a^low^ group	PRODH2	PRODH2 enhances the metabolic and immune functions of CAR-T cells against cancer	[41]
CRISPR KO	Primary human CD4^+^ T cells	in vitro	GPCR’s gene library	TCR activation and sorting of CD69, IL-2 and IFN-γ expression by FACS	Compare the the high and low surface expressions of CD69, the production of IL-2 and IFN-γ	S1PR1, GPR183	Contribute to T cell activation	[51]
CRISPR KO	Jurkat T cells	in vitro	Genome-wide libraries	FASL stimulation	Compare the control group and the FASL stimulation group	EBF4	EBF4 modulates FAS-mediated apoptosis and promotes cytotoxic function	[52]
shRNA	CD8^+^ T cells	in vitro	shRNA libraries associated with chromatin	FACS sorts CD4 expression	Compare CD4^+^ group and CD4^−^ group	CAF-1	CAF-1 stably repress expression of Cd4 gene	[53]
CRISPRa and CRISPRi	Primary human CD8^+^ T cell	in vitro	A gene library of epigenetic modifiers associated with T cell status	FACS sorts CCR7 expression	Compare CCR7^high^ group and CCR7^low^ group	BATF3	BATF3 promotes specific features of memory T cells	[54]
CRISPR KO	OT-I T cells	In vivo	Metabolism-related gene libraries	Injected into mice, FACS sorts KLRG1^−^CD127^+^ (MP) and KLRG1^+^CD127^−^ (TE) phenotypes	Compare the MP group, TE group and non-injected cell group	Slc7a1, Slc38a2	Slc7a1 and Slc38a2 dampene the magnitude of T_MEM_ differentiation through modulating mTORC1 signaling	[57]
CRISPR KO	OT-I T cells	in vivo	A library that targets proteins involved in epigenetic modifications	Injected into mice, FACS sorts TE, MP, and normal phenotypes	Compare the MP group, TE group and non-injected cell group	cBAF	cBAF inhibit T_mem_ cells formation	[56]
CRISPR KO	OT-I T cells	in vivo	Genome-wide libraries	TCR was repeatedly stimulated to observe T cell changes	Compare the IL-2 stimulation alone group and the α-CD3 and IL-2 stimulation group	INO80, Arid1a	INO80 and BAF chromatin inhibit remodeling complexes improved T cell persistence	[59]
Single-cell CRISPR KO	OT-I T cells	in vivo	Transcription factor gene library	B16-OVA mice were injected to observe T cell survival	Direct sequencing reads after 7 days	IKAROS ETS1	The IKAROS–TCF-1 axis in Tpex cell quiescence exit and the ETS1–BATF axis limits Tex cell generation	[30]
CRISPR KO	CD8^+^ P14 cells	in vitro	TF library	Co-cultured with CD11C^+^DC cells for acute and chronic GP33 and IL-2 stimulation	Compare T cells on the second day versus the seventh day	BHLHE40	BHLHE40 regulates T cell exhaustion	[60]
CRISPR KO	Primary human CD8^+^ T cell	in vitro	Epigenetic Regulator Library	Acute and chronic TCR stimulation was performed in vitro, and FACS sorted PD1^+^/TIM3^+^ expression	Compare Acute and chronic stimulation groups	mSWI/SNF	mSWI/SNF family chromatin remodeling complexes direct T cell activation and exhaustion	[61]
CRISPR KO	68–41 T cells	in vitro	Genome-wide libraries	After in vitro culture, FACS sorts PD-1 expression	Compare PD-1^high^ group and PD-1^low^ group	Fut8	Fut8 induce cell-surface expression of PD-1 and T cell exhaustion	[62]

*St3gal1* ST3 β-galactoside α-2,3-sialyltransferase 1, *RASA3* Ras gtpase activating proteins3, *RASA2* Ras gtpase activating proteins2, *NAMPT* nicotinamide phosphoribosyltransferase, *LTBR* lymphotoxin-β receptor, *P38* a class of mitogen-activated protein kinases, *FAM49B* family with sequence similarity 49 member B, *PIK3CD* phosphoinositide-3-kinase catalytic subunit delta, *DHX37* DEAH-Box Helicase 37, *PRODH2* Proline Dehydrogenase 2, *S1PR1* sphingosine-1-phosphate receptor, *GPR183* G Protein-Coupled Receptor 183, *EBF4* early B cell factor 4, *CAF-1* chromatin assembly factor, *BATF3* Basic leucine zipper transcription factor ATF-like 3, *Slc7a1 and Slc38a2* amino acid transporters, *cBAF* mammalian canonical Brg1/Brg-associated factor, *INO80* INO80 Complex ATPase Subunit, *Arid1a* AT-Rich Interaction Domain 1A, *IKAROS* Family Zinc Finger Protein 2, *ETS1* E-twenty six1, *BHLHE40* Basic Helix-Loop-Helix Family Member E40, *mSWI/SNF* mammalian SWItch/Sucrose Non-Fermentable, *Fut8* Fucosyltransferase 8

Due to challenges like insufficient targeted delivery efficiency, solid tumor barriers, and organ aggregation, intravenously administered adoptive T cells often fail to fully target tumor cells, leading to reduced therapeutic effects and acute toxicity [44]. For example, Hong and colleagues found that CD8^+^ T cells with an sgRNA library, when implanted into mice, exhibited enhanced homing to tumor tissues with the overexpression of St3gal1 and βII-spectrin [45]. Additionally, integrin lymphocyte function-associated antigen 1 (LFA-1) facilitates T cell migration and adhesion by binding to intercellular adhesion molecule 1 (ICAM-1). Screening for ICAM-1^pos^ and ICAM-1^neg^ cells identified RASA3 as a gene that enhances the migratory efficiency of adoptive T cell therapy [46]. T cell proliferation is indicated by carboxyfluorescein succinimidyl amino ester (CFSE) dye activity. Selecting T cells with high proliferation rates and validating them in CAR-T cells allows for the identification of active positive regulatory genes [12, 27, 47, 48]. For example, CD19 CAR-T cells with RASA2 knockout demonstrated enhanced adaptability and effector functions in a B-cell acute lymphoblastic leukemia (B-ALL) mouse model [12]. CD69, an early-expressed molecule in lymphocyte activation, helps identify modulators of T cell activity. Sorting CD69^high^ and CD69^low^ cell groups via FACS revealed that FAM49B inhibits T cell activation by modulating Rac activity and cytoskeletal reorganization [49].

Functional activation sites in T cells can be identified by targeting cytokines such as interferon-γ (IFN-γ) or CD8^+^CD107a^+^ T cells [36, 41, 50, 51]. For example, Ye et al. identified GOF targets like Proline Dehydrogenase 2 (PRODH2) in CD8^+^CD107a^+^ T cells through CRISPRa screening. Integrating PRODH2 into primary or CAR-T cells reshapes metabolic pathways, enhancing outcomes across diverse tumor types [41]. Advanced base editing screening has also revealed alleles, such as those encoding the phosphoinositide-3-kinase catalytic subunit delta (PIK3CD) protein, that modulate T cell cytotoxic functions in both positive and negative ways [36]. In T cell apoptosis, Fas-FasL (Factor-related Apoptosis ligand) signaling is significant. Continuous FasL stimulation compared to normal controls showed that Early B cell factor 4 (EBF4) regulates the degradation of anti-apoptotic cellular FLICE-inhibitory protein (c-FLIP), inducing the dissolution of cytotoxic T cells [52]. Various gene expression programs facilitate the induction and maintenance of diverse T cell subsets in adaptive tumor immunity. CD4^+^ and CD8^+^ single-positive cells require several rounds of selective differentiation. For instance, shRNA screening targeting CD4 expression has shown that chromatin assembly factor (CAF-1) and DNA methyltransferase collaborate to silence the CD4 gene, maintaining the CD8^+^ T cell phenotype [53]. Furthermore, studies indicate that CD8^+^ T cell immunity depends on differentiation into effector (T_EFF_) and memory (T_MEM_) cells. C–C motif chemokine receptor (CCR7) and CD62L are recognized T_MEM_ surface markers, aiding in identifying regulators of T_MEM_ differentiation and maintenance [48, 54]. For example, CRISPRa and CRISPRi screening of CCR7 expression revealed that Basic Leucine Zipper ATF-Like Transcription Factor 3 (BATF3) overexpression boosts specific T_MEM_ traits, enhancing HER2^+^ CAR-T efficacy against breast cancer [54]. Short-term effector cells (TE; KLRG1^+^ CD127^−^) and memory precursor cells (MP; KLRG1^−^ CD127^+^) are distinguishable, with TEs perishing within days to weeks, while MPs evolve into T_MEM_s and provide prolonged protective tumor immune responses [55]. Comparing MP/TE post-cultivation in mice identifies regulatory factors associated with a more memory-like phenotype, shaping the destiny of CD8^+^ T cells [56, 57].

Many patients struggle with long-term responses to adoptive T cell immunotherapy, often facing drug resistance or relapse post-treatment. Identifying factors influencing T cell functional persistence is critical. T cell exhaustion, characterized by diminished effector capacity and heightened immune response suppressor (IRS) expression, is a key factor [58]. High-throughput genetic screening can discern persistence factors by repeatedly stimulating T cells with anti-CD3/CD28 and IL-2 in vitro, or by implanting them into tumor-bearing mice and comparing chronically stimulated T cells with unstimulated counterparts [30, 59–61]. Examining regulators of exhaustion, such as characteristic IRS like PD-1, involves screening, reading, and validation processes. Techniques such as gene ablation or pharmacological inhibition of core fucosylation (Fuco) to reduce PD-1 expression can enhance the anti-tumor effectiveness of adoptive T cell therapy [62].

### TCR-T and CAR-T cell directly screening

Direct screening within TCR-T and CAR-T cells is an emerging area of research with limited exploration to date. Typically, engineered TCR or CAR sequences are introduced into activated ex vivo primary T cells using retrovirus or lentivirus vectors. Similar to primary T cell screening, sgRNA or other gene-editing libraries are introduced into TCR-T or CAR-T cell pools. The cytotoxicity of adoptive T cells is assessed by co-culturing with tumor cells, followed by direct sequencing of groups with high or low functional cytokine expression or PD-1^+^ and PD-1^−^ cell groups to identify factors affecting TCR-T and CAR-T cell cytotoxic function or persistence. Researchers employ FACS or scRNA-seq to isolate T cell subpopulations and identify functional genes through sequencing. FACS uses fluorescent labeling and voltage adjustments to isolate cells based on specific markers like functional cytokines and PD-1, followed by conventional sequencing to pinpoint functional genes. scRNA-seq enables direct isolation and high-throughput sequencing of individual T cells, providing more accurate identification of functional genes across diverse T cell populations.

Researchers focus on enhancing the expression of cytotoxicity-associated molecules while suppressing exhaustion-related factors in T cells to amplify T cell activation signals and overall functionality. For instance, Freitas et al. isolated GD-2 CAR-T cells co-cultured with tumor cells based on cytotoxic cytokine expression (TNF and IL-2) and identified MED12 and CCNC, components of the mediator cell cycle protein-dependent kinase module, as targets that enhanced anti-tumor immunity through transcriptional and epigenetic changes [63]. Furthermore, the longevity of T cells can be extended by isolating cell populations exhibiting downregulation of T cell exhaustion-associated proteins. Wang and team used CRISPR screening of PD1^+^ HER2^+^ CAR-T cells and glioblastoma stem cells to find that knocking out Transducin-Like Enhancer of split 4 (TLE4) reduced apoptotic traits, sustaining CAR-T cell function, while ablation of Ikaros family zinc finger protein 2 (IKZF2) improved CAR-T anti-tumor efficacy by regulating NFAT signaling [64]. NY-ESO-1 TCR-T cells generated from CD8^+^ T cells revealed that Subsequent investigations revealed that sorting nexin 9 (SNX9) regulates both T cell exhaustion (PD1^+^) and cytotoxic function (CD107a^+^), with SNX9 knockout enhancing T cell activation signals and anti-tumor potential [65]. Direct screening within TCR-T and CAR-T cells not only intuitively discovers adoptive T cell therapy’s regulatory mechanisms but also utilizes engineered TCR and CAR molecules in T cells for heightened tumor-targeting cytotoxicity. This method improves the efficacy of tumor cell co-culture screening and more readily establishes continuous tumor antigen stimulation models, advancing adoptive T cell therapy.

**Fig. 3:**
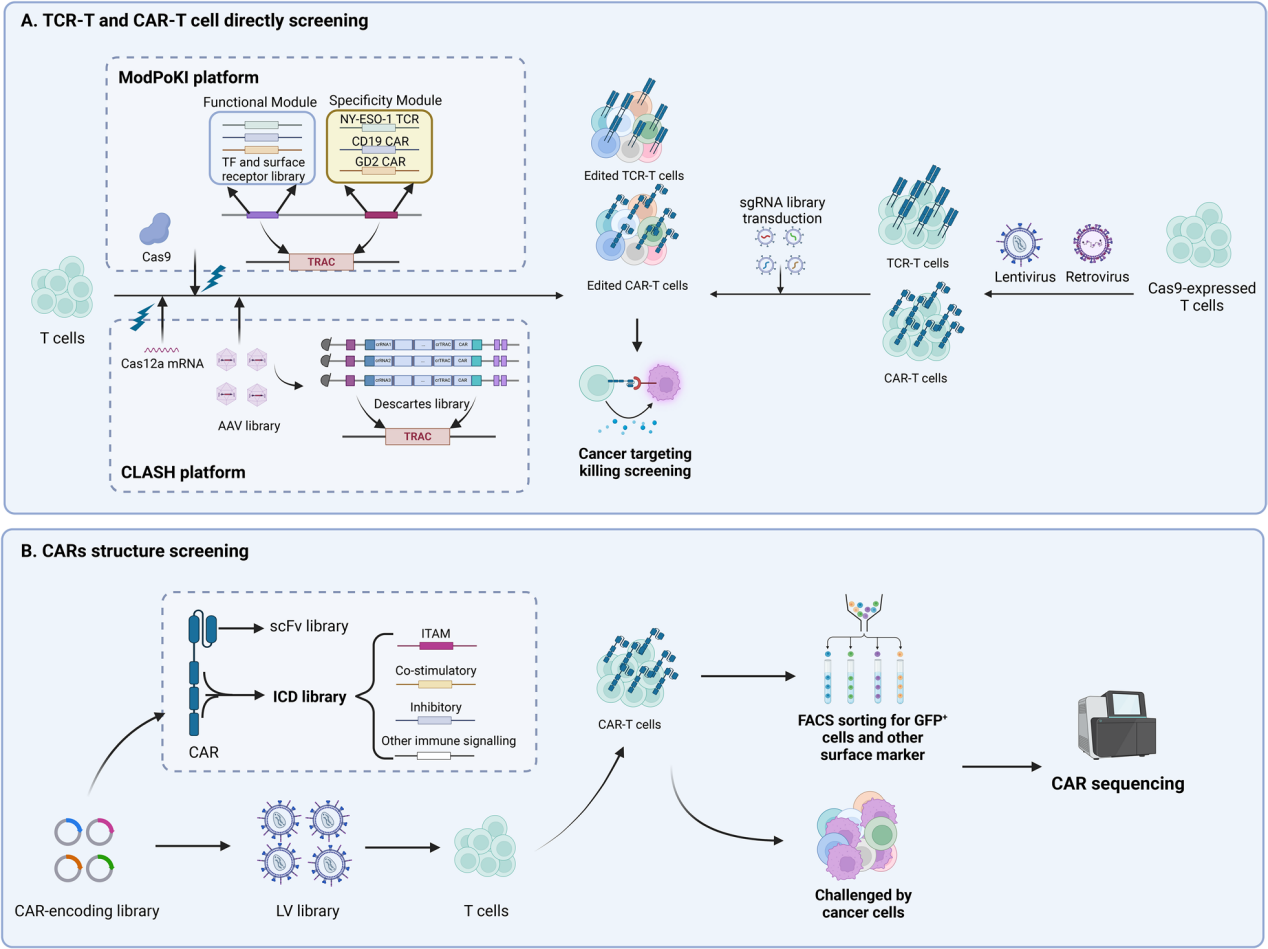
Adoptive T cell directly screening and CARs structure screening. **A** In the context of TCR-T and CAR-T cell therapies, introducing TCR and CAR constructs into T cells via lentiviruses and leveraging the ModPoKI platform enables the co-targeting of sgRNA and Cas9, equipped with Functional and Specificity Modules, to the TRAC locus through electroporation. This approach facilitates the generation of TCR-T and CAR-T knock-in libraries. Concurrently, the CLASH platform’s CRISPR KI/KO AAV library allows for simultaneous CAR integration and genetic modification at the TRAC locus in CAR-T cells, minimizing potential inaccuracies caused by the use of dual viral vectors. The subsequent co-culture of these genetically engineered cells with tumor cells enables the direct selection of effective TCR-T and CAR-T candidates based on their tumoricidal activity. **B** The architecture of CARs principally comprises the scFv and the ICD. Constructing an ICD library incorporating motifs such as ITAMs, co-stimulatory, and inhibitory domains, followed by lentiviral transduction into T cells, allows for the selection of CAR-T cells based on criteria including proliferation, differentiation, cytotoxicity, and durability. Sequencing of CARs then facilitates the identification of the most effective CAR-T configurations

Direct screening in TCR-T and CAR-T cells offers intuitive insights into adoptive T cell therapy’s regulatory mechanisms, improving tumor-targeting cytotoxicity by utilizing engineered TCR and CAR molecules. However, challenges include reduced efficiency of subsequent viral transductions for gene editing due to pre-transduction of engineered sequences, which can disrupt target genes and complicate data interpretation [9]. To address these issues, Dai et al. developed the CLASH system using AAV-KIKO and CRISPR/Cas12a, where Cas12a mRNA is introduced into human T cells, and a crRNA library targets both CAR sequences and the T Cell Receptor Alpha Constant (TRAC) locus. This approach enables high-throughput CRISPR KO screening and CAR knock-in at the TRAC locus, overcoming the limitations of traditional CAR gene transduction methods. Utilizing CLASH, they identified the exon3 skip mutant of PR/SET Domain 1(PRDM1) as significantly boosting anti-tumor effects in CD22 CAR-T cells [66]. Similarly, Blaeschke et al. introduced the Modular Pooled Knock-In (ModPoKI) approach, attaching functional modular libraries to sgRNA for TCR-T and CAR-T production at the TRAC locus, revealing that overexpression of BATF and transcription factor AP4 (TFAP4) jointly influences TCR/CAR gene expression and T cell functionality in anti-tumor immunity [67] (Table 3 and Fig. 3A).

**Table 3:** Representative Studies of Genetic Screening for TCR-T and CAR-T cells

Screening methods	Screening cells	Screening model	Construction of TCR-T or CAR-T	Library design	Screening strategies	Comparison and readout	Screened targets	Function of Screened target	Ref
CRISPR KO	NY-ESO-1 TCR-T cells	in vitro	Lentivirus transduction	Cytotoxic T cell gene pool	TCR stimulation, FACS sorting CD107a expression	Compare CD107a^+^ group and CD107a^−^ group	SNX9	Deletion of SNX9 alleviates CD8 T cell exhaustion	[65]
CRISPR KO	HER2 CAR-T cells	in vitro	Lentivirus transduction	Genome-wide libraries	Co-cultured with Glioblastoma stem cells, FACS sorts PD1 expression	Compare of PD1^+^ and PD1^−^ groups	TLE4, IKZF2	TLE4 and IKZF2 induce exhaustion responses through transcriptional process	[64]
CRISPR KO	GD2 CAR-T cells	in vitro	Retrovirus transduction	Genome-wide libraries	Co-cultured with GD2^+^ tumor cells, FACS sorted TNF and IL-2 expression	Compare TNF^±^ with IL-2^±^ groups	MED12	MED12 deletion enhanced antitumor activity and sustained the effector phenotype in CAR- T cells	[63]
CRISPR KO	CD22 CAR-T cells	in vitro in vivo	CLASH platform	Descartes library	Co-cultured with the NALM6 cell line NALM6-GL mice were injected into the body, and the changes of T cells over time were observed	Compare T cells over three time periods	PRDM1	The PRDM1 Δexon3 mutant improves the persistence of CAR-T cell	[66]
CRISPR KI	NY-ESO-1 TCR-T cells, CD19 CAR-T cells	in vitro	ModPoKI platform	TF library and surface receptor library	TCR was repeatedly stimulated to observe T cell changes	Compare T cells over different time periods	BATF, TFAP4	Overexpressed BATF and TFAP4 improves the persistence of T cells	[67]

*SNX9* sorting nexin-9, *TLE4* Transducin Like Enhancer of Split 4, *IKZF2* Ikaros Family Zinc Finger Protein 2, *MED12* Mediator Complex Subunit 12, *PRDM1* PR/SET Domain 1, *BATF* Basic Leucine Zipper ATF-Like Transcription Factor, *TFAP4* Transcription Factor AP-4

### CARs structure screening

The specificity of CAR-T and TCR-T therapies is derived from the targeted recognition between tumor cell surface antigens and the engineered antibodies or TCRs on T cells. Identifying suitable target antigens remains a significant challenge in engineered adoptive T cell therapy. The tumor microenvironment (TME) often inhibits T cell infiltration and may lack tumor-specific antigens, leading CAR-T therapies to target tumor-associated antigens (TAAs). However, TAAs can also be present in normal tissues, which may result in unpredictable on-target, off-tumor (OTOT) effects [68]. Consequently, optimizing CAR structures to enhance affinity is a promising strategy. By utilizing CAR structure screening, researchers can identify constructs with improved specificity and affinity for target antigens, thereby gaining deeper insights into the functional and mechanistic differences among CAR structures. By modulating CAR affinity, CAR-T cells can be engineered to better differentiate between normal cells with low antigen density and tumor cells with higher antigen density. This approach may enhance CAR-T cell functionality while minimizing off-target effects, toxicity, and side effects, ultimately improving CAR-T cell durability. Researchers can also optimize CARs with varying molecular backgrounds by screening different modules of CAR structures to meet diverse clinical needs, establishing a foundation for the discovery of next-generation CARs [69–72].

CARs are composed of three main components: the extracellular single-chain variable fragment (scFv), the hinge and transmembrane domain, and the intracellular signaling domain. The scFv component, which targets the antigen, is introduced into CAR structures via lentivirus, with extensive scFv production achieved through “light chain shuffling.” Methods of affinity screening and efficacy assessment evaluate the affinity, proliferation, differentiation, and tumor-killing potential of CD38 CAR-T cells. It was found that reducing the affinity of CD38-CAR T cells by approximately 1,000-fold was most effective at lysing CD38^+^ multiple myeloma (MM) cells [72]. Additionally, a Combinatorial Cellular Library of CD38 CARs enables the enrichment of specific CD38 subgroups through negative screening with normal tissue cells and positive screening with tumor cells, revealing the highest affinity CAR structure designs [73]. Moreover, modifying the complementary determining region of HER2^+^ CAR-T’s variable heavy chain, while monitoring CAR expression, antigen recognition, and signal transduction, demonstrates the importance of CAR structural design in affecting affinity and cytotoxicity, thereby reducing OTOT effects [74].

Intracellular domains (ICDs) are composed of various signaling modules that convert antigen recognition into downstream anti-tumor actions. These domains are crucial in orchestrating CAR-T cell behavior and primarily include basic modules of CD3ζ’s immunoreceptor tyrosine-based activation motif (ITAM), co-stimulatory domains like CD28 and 4-1BB, as well as other activating or inhibitory signals [75]. Different combinations of these signaling domains can significantly influence T cell therapeutic efficacy. However, designing and testing each CAR-T construct individually is labor-intensive. To address this, researchers introduce an orthogonally-designed CAR library—which includes diverse ITAMs, co-stimulatory domains, inhibitory signals, and other immune signals—into T cells via lentivirus at a consistent locus, creating a large CAR-T cell pool. Assessment and selection are based on cell proliferation, differentiation, cytotoxicity, and persistence. Sequencing these CAR constructs helps confirm the optimal functionality of the CAR-T cells [71, 76–78]. Gorden and his team employed the CARPOOL strategy to swiftly enrich and identify novel CD19 CARs with clinically valuable phenotypes. They found that the Var1 CAR outperformed the widely-used BBζ CAR in vitro, although both exhibited comparable efficacy in vivo [78]. Additionally, the speedingCARs platform was developed, combining HER2^+^ CAR signal domain recombination with collective functional screening. This approach revealed the impacts of CAR variations through scRNA-seq and single-cell CAR sequencing [76]. Establishing similar platforms can help overcome the limitations of CAR-T therapy and be extended to include TCR-T and other cellular therapies, such as NK cells and macrophages (Fig. 3B).

Current CAR structure screening primarily relies on immortalized cell lines, which display altered baseline signaling and metabolism. As a result, certain T cell functions, such as anti-exhaustion and effective tumor killing, may not be accurately reflected. Furthermore, there is significant heterogeneity in CAR structure screening, with in vivo improvements often being less pronounced than those observed in vitro. In vitro models may amplify differences in antigen exposure abundance and kinetics, overlooking the impact of the immune microenvironment. Additionally, the scope of current CAR structure screening is narrow, and the efficacy of screened CAR structures across different tumors is not yet clear, with insufficient exploration of their associated safety. Significant challenges remain in translating novel CAR structures identified through CAR structure screening into clinical applications.

## Genetic screening applied for tumor cells under T cell pressure

Tumor cells commonly evade adoptive T cell immunotherapy due to various factors such as antigen downregulation, reduced cytokine sensitivity, and metabolic alterations, which contribute to resistance and relapse in T cell-focused treatments [79]. High-throughput genetic screening serves as an impartial platform for target discovery, offering a thorough method to characterize tumor cell and T cell interactions. Applying T cell pressure to tumors can mimic adoptive T cell immunotherapy and is crucial for identifying new therapeutic targets or potential regulatory mechanisms to improve efficacy. Similar to T cell screening, tumor cells, after introducing sgRNA libraries and other gene-editing tools, are co-cultured in vitro with T cells or CAR-T cells. These are compared to cells without survival stress, or direct data readouts from surviving tumor cells are analyzed to identify genes linked to drug resistance. In in vivo screening, tumor cells can be transplanted into both immunosuppressed and immunocompetent mice, facilitating a deeper analysis of key regulatory mechanisms in tumor immune infiltration. This process allows for the validation and enhancement of adoptive T cell therapies using small molecule drugs (Table 4).

**Table 4:** Representative Studies of Genetic Screening for tumor cells under T cell pressure

Screening methods	Screening cells	Screening model	Library design	Screening strategies	Comparison and readout	Screened targets	Function of Screened target	Ref
CRISPR KO	MHC I Negative K-562 cells	in vitro	220 k sgRNA library	Three rounds of FACS Sorting MHC I expression	Compare the MHC I^pos^ group and MHC I^neg^ group	PRC2	PRC2 silences the MHC I antigen presentation pathway and enables immune evasion	[80]
CRISPR KO	MHC II Negative MOLM13 and OCI-AML3 cells	in vitro	15.3 k or 220 k sgRNA library	Three rounds of FACS Sorting MHC II expression	Compare the MHC II^pos^ group and MHC II^neg^ group	CtBP, FBXO11	Inhibition of the CtBP complex and FBXO11 enhances MHC class II expression and anti-cancer immune responses	[81]
CRISPR KO	A357 cells	in vitro	Genome-wide libraries	CD58 staining, FACS sorting CD58 expression	Compare CD58^mi^ group and CD58^lo^ group	CMTM6	CMTM6 is critical for CD58 stability and upregulation of PD-L1 upon CD58 loss	[89]
CRISPR KO	BCP-ALL cells	in vitro	Genome-wide libraries	After in vitro culture, FACS sorting CD19 expression	Compare the CD19^high^ group and CD19^low^ group	ZNF143, NUDT21	ZNF143 promotes CD19 activation and NUDT21 limits expression of CD19	[87]
CRISPRa and CRISPRi	AMO1 cells	in vitro	Genome-wide libraries	Anti-BCMA antibody stimulation, FACS sorting BCMA expression	Compare the BCMA^high^ group and BCMA^low^ group	HDAC, Sec61	Inhibition of HDAC7 and the Sec61 complex increased cell surface BCMA	[86]
CRISPR KO	Daudi cells	in vitro	Genome-wide libraries	Co-cultured with Vγ9Vδ2 T cells to observe cancer killing	Compare the killed cells and survival cells	AMPK	Activation of AMPK led to increased expression of the BTN2A1– BTN3A complex and increased Vγ9Vδ2 T cell receptor-mediated killing	[85]
CRISPR KO	Colorectal cancer cells	in vitro	Genome-wide libraries	IFN-γ stimulation, FACS sorting MHC-I and PD-L1 expression	Compare the stimulation group and the untreated group	JAK1	Mutations in JAK1 enhanced or reduced sensitivity to autologous tumor-reactive T cells	[38]
CRISPR KO	B16-F10 cells	in vitro	Genome-wide libraries	IFN-γ and TNF overnight culture to observe cancer cell changes	Compare cancer cells over different time periods	HOIP	Inhibition of HOIP promote tumor cell sensitivity to TNF and IFN-γ	[92]
CRISPR KO	Melanoma cells	in vitro	Genome-wide libraries	Antibody Staining, FACS sorting IFN-γR1 expression	Compare IFN-γR1^high^ group, IFN-γR1^low^ group, and the untreated group	STUB1	STUB1 inactivation amplifies IFN-γ signaling, sensitizing tumor cells to cytotoxic T cells	[94]
CRISPR KO	KPC3-OVA cancer cells	in vitro	Genome-wide libraries	Co-cultured with OT-I T cells to observe cancer killing	Compare Epi cancer cells and Mes cancer cells	Egfr, Mfge8	Egfr and Mfge8 are significantly higher expressed in Mes cancer cells, and their depletion sensitized Mes cancer cells to CTL-mediated killing	[118]
CRISPR KO	AML3 cells	in vitro	Epi-drug library	Co-cultured with DNT cells, FACS sorting Annexin V	Compare killed cells and survival cells	CD64	The level of CD64 expression correlated strongly with the sensitivity of AML cells to DNT treatment	[119]

*KO* Knock out, *MHC* Major histocompatibility complex, *FACS* Fluorescence activated Cell Sorting, *PRC2* polycomb repressive complex 2, *CtBP* C-terminal binding protein, *CMTM6* CKLF-like MARVEL transmembrane domain-containing protein 6, *ZNF 143* Zinc Finger Protein 143, *NUDT21* Nudix Hydrolase 21, *BCMA* B-cell maturation antigen, *HDAC* Histone deacetylase, *AMPK* AMP-activated protein kinase, *BTN2A1* Butyrophilin 2A1, *IFN-γ* Interferon-γ, *TNF* Tumor necrosis factor, *Epi* Epithelial–like, *Mes* Mesenchymal-like, *Egfr* epidermal growth factor receptor, *Mfge8* Milk Fat Globule-EGF Factor 8 Protein, *DNT* Double Negative T

### Targeted antigen presentation

In adoptive cell therapies, particularly engineered TCR-T and CAR-T therapies, specific recognition between tumor cell surface antigens and engineered TCRs or antibodies on T cells is essential. Enhancing antigen presentation and preventing antigen downregulation are vital strategies to mitigate resistance. After Major Histocompatibility Complex (MHC) antibody stimulation or co-culturing, direct screening based on the expression of MHC molecules or other TAAs is performed. Sequencing following FACS sorting reveals regulatory factors linked to antigen expression, both positive and negative [80–87].

Researchers conducted gene editing on MHC-I^neg^ cells using an sgRNA library. After three rounds of FACS selection for MHC-I^pos^ cells, they identified PRC2-mediated transcriptional silencing in the MHC-I antigen processing pathway (MHC-I APP). PRC2-mediated silencing of MHC-I drives resistance of small cell lung cancer to T cell-mediated killing and promotes the spread of non-tissue-compatible receptors, thereby facilitating T cell immune evasion. Genetic disruption or pharmacological inhibition of PRC2 can restore tumor MHC-I antigen presentation, enabling effective targeting by CD8^+^ T cells and enhancing tumor immunity. While this study's data primarily highlight the role of PRC2 in MHC-I antigen presentation, other candidate genes identified through screening remain. Future research should explore the roles of additional transcription factors in regulating antigen presentation [80]. Similarly, MHC-II^pos^ screening revealed that the CtBP complex transcriptionally represses the MHC II presentation pathway. Targeting these inhibitory mechanisms can selectively induce the upregulation of MHC class II in various AML cell lines, thereby stimulating antigen-dependent CD4^+^ T cell activation and promoting an effective anti-tumor immune response [81]. Additionally, the tumor cell surface protein CD58 binds to CD2 receptors on T cells. Post-TCR and MHC antigen presentation, this binding delivers co-stimulatory signals. CD58 serves as an adhesion molecule, enhancing the initial binding of effector T cells [88]. Sorting CD58^high^ and CD58^low^ tumor cells revealed that CKLF-like MARVEL Transmembrane Domain Containing 6 (CMTM6) synergistically regulates co-stimulatory (CD58) and co-inhibitory (PD-L1) signals in cancer cells. Both CD58 and PD-L1 may need to bind directly to the extracellular loop of CMTM6. In the absence of CD58 expression, additional PD-L1 protein binds to the released CMTM6 and stabilizes it on the cell surface by enhancing circulation and reducing lysosomal degradation, potentially decreasing resistance to immune checkpoint blockade (ICB) therapy. CMTM6 serves as a crucial regulator of co-stimulatory and co-inhibitory signals in cancer cells. However, further research is necessary to elucidate the structure of CMTM6 and determine its precise interactions with CD58 and PD-L1, providing better insights for therapeutic design [89].

In CAR T-cell therapy, CD19 is a prevalent target for blood cancer treatment. Screening CD19^+^ expression in B cell precursor acute lymphoblastic leukemia (BCP-ALL) revealed the contrasting functions of Zinc Finger Protein 143 and Nudix Hydrolase 21 (NUDT21). The absence of NUDT21 in BCP-ALL cells enhances CD19 expression, increasing susceptibility to CD19-specific CAR-T [87]. B cell maturation antigen (BCMA) CAR-T therapy for MM also employs CRISPRa screening to pinpoint regulators of antigen presentation and loss [86]. For particular lymphocytes like γδ T cells, which mostly function in innate immunity without needing MHC pathway activation, Vγ9Vδ2 T cells are the predominant subgroup. They recognize complexes with butyrophilin Subfamily 2 Member A1 (BTN2A1) and butyrophilin Subfamily 3 Member A1. Enhanced BTN3A expression on tumor cells can activate γδ T cell cytotoxicity, providing a novel method to boost γδ T cell anti-cancer activity and to develop γδ T cell-based therapies [85].

### Intrinsic mechanisms of cytokine resistance

Beyond antigen downregulation, tumor cells exhibit various other immune escape and resistance mechanisms. A prevalent mechanism is their inherent insensitivity to cytokines secreted by cytotoxic T cells, such as IFN-γ and TNF. IFN-γ enhances immune infiltration by secreting chemokines that attract lymphocytes, aiding in tumor antigen presentation and processing, and increasing tumor cell sensitivity to destruction. Consequently, abnormalities in the IFN-γ-JAK-STAT pathway can lead to resistance [90]. TNF-mediated nuclear factor kappa-B signaling exerts inhibitory effects on tumor cells or enhances the migration and infiltration of activated immune cells, working alongside IFN-γ to achieve anti-tumor effects [91]. Continuous stimulation of sgRNA-transduced tumor cells with IFN-γ or TNF allows for the identification of regulatory factors that are sensitive or resistant to these cytokine pathways, based on the survival of the tumor cells [38, 92, 93]. Coelho et al. used base editing screening to identify mediators of sensitivity or resistance to IFN-γ in colorectal cancer, revealing hundreds of missense mutations that can predict alterations in IFN-γ pathway activity [38]. Freeman et al. found that the LUBAC catalytic subunit HOIP inhibits both intrinsic and extrinsic apoptotic pathways, thereby diminishing tumor immunity, through screening with combined stimulation of IFN-γ and TNF [92]. Additionally, Apriamashvili et al. identified that the E3 ubiquitin ligase STUB1, which negatively regulates IFN-γ signaling, is a mechanism of tumor cell resistance. This was achieved by sorting the top 10% and bottom 10% of IFN-γ receptor 1 (IFN-γR1) melanoma cells using FACS, broadening research on the IFN-γ pathway from the receptor level [94].

Comparing tumor and T cell co-cultures in vitro and in vivo, analyzing the effects of treatment on gene expression, or contrasting situations in immunocompetent or deficient mice, researchers use GSEA or functional verification experiments to search for or validate upstream factors of targeted antigen presentation and intrinsic mechanisms of resistance. Identified targets reflect tumor cell sensitivity or resistance under T cell pressure, typically affecting antigen presentation, IFN and TNF signaling, and cytotoxic T cell killing functions. This common research model has achieved results in primary T cells [95–112] and CAR-T cells [113–116]. For example, the creation of the immune escape cancer cell line KPIE2, in co-culture with gp100 TCR-T, involved CRISPRa screening of melanoma cells' lncRNAs. GSEA analysis identified IL10RB Divergent Transcript (IL10RB-DT) as an inhibitor of antigen presentation and IFN-γ-JAK-STAT1 signaling [95]. In a lung cancer mouse model, introducing sgRNA alongside ACT revealed the testicular cancer antigen ADAM Metallopeptidase Domain 2 (ADAM2) as an immune modulator. ADAM2 reduces tumor antigen expression and impedes the Type I and II IFN and TNFα pathways, contributing to the establishment of a cold TME. However, it suppresses immune checkpoint molecules PD-L1, Lymphocyte Activating 3, and T Cell Immunoreceptor With Ig And ITIM Domains, thereby enhancing the efficacy of adoptively transferred cytotoxic T cells in tumors overexpressing ADAM2 [100]. Co-culturing Epidermal Growth Factor Receptor (EGFR) CAR-T cells with glioblastoma cells transfected with sgRNA for CRISPR screening demonstrated that the IFN-γ-R signaling pathway confers greater resistance to CAR-T cell killing in glioblastoma and other solid tumors, but not in leukemia and lymphoma, indicating distinct effects between solid and hematologic malignancies [113].

### Other mechanisms and strategies for tumor cells

The epithelial-mesenchymal transition (EMT) represents a novel mechanism of tumor escape, characterized by epithelial (Epi) cancer cells losing intercellular adhesion and transforming into mesenchymal-like (Mes) cancer cells. This change facilitates migration and invasion, thereby enhancing resistance to immunotherapies [117]. In vitro, the EMT system, induced by transforming growth factor-β and Doxycycline, yields tumor cells with distinct Epi or Mes characteristics. Upon sgRNA transfection and co-culturing with CD8^+^ T cells, EGFR and Milk Fat Globule-EGF Factor (Mfge8) emerged as Mes-specific cytotoxic regulators that facilitate immune escape in CD8^+^ T cells, highlighting compelling pharmacological targets [118].

DNTs (TCR^+^CD3^+^CD4^−^CD8^−^) are peripheral mature T cells that do not provoke host anti-graft or graft-versus-host reactions, making allogeneic DNTs a promising candidate for "off-the-shelf" adoptive T cell therapy. Co-culture screening identified that components of the SPT-ADA-GCN5 acetyltransferase deubiquitination complex and CD64, respectively, downregulate and upregulate DNT cytotoxicity against AML, thereby improving the efficacy of DNT therapy [119].

## Genetic screening applied for promising combination strategies

### Immune checkpoint blockade

ICB therapy inhibits the IRS, leading to the reversal of T cell exhaustion and the restoration of T cell infiltration into tumors. It also blocks checkpoint ligands between tumor cells and other antigen-presenting cells (APCs), thereby facilitating T cell signal transduction and cytotoxicity. Clinical evidence has shown that blockade of the PD-1/PD-L1 pathway and Cytotoxic T-Lymphocyte-Associated protein 4 (CTLA-4) leads to sustained anti-tumor responses, significantly altering treatment outcomes in advanced and metastatic cancers. This signifies a conventional T-cell mediated approach in tumor immunotherapy [120]. However, the majority of patients exhibit limited response to checkpoint immunotherapy and develop resistance following treatment, making it crucial to thoroughly investigate the mechanisms regulating ICB efficacy [121]. In high-throughput genetic screening, the ICB treatment process in cancer patients is simulated by implanting gene-edited tumor cells in mice and administering anti-PD-1 or anti-CTLA-4 intravenously. This method, using sgRNA readouts, identifies genes that act as drivers or inhibitors in ICB treatment [122–126]. For instance, introducing an sgRNA library into various tumor cells and categorizing them into in vitro cultures, immunodeficient NSG mice, wild-type mice, and ICB treatment groups, reveals that IFN-induced non-classical MHC class I molecules, Qa-1b/HLA-E, can impede ICB treatment [122]. Additionally, intrinsic Anti-Silencing Function 1A Histone Chaperone (ASF1A) deficiency in tumor cells can enhance anti-PD-1 therapy efficacy by upregulating Granulocyte–Macrophage Colony-Stimulating Factor (GM-CSF) expression [126]. For instance, introducing an sgRNA library into various tumor cells and categorizing them into in vitro culture, immunodeficient NSG mice, wild-type mice, and ICB treatment groups, reveals that IFN-induced non-classical MHC class I molecules, Qa-1b/HLA-E, can impede ICB treatment [122]. Additionally, intrinsic ASF1A deficiency in tumor cells can enhance anti-PD-1 therapy efficacy by upregulating GM-CSF expression [126].

Upregulation of PD-L1 in tumor cells, which facilitates escape from T-cell-based immunotherapy, is a potential mechanism behind resistance to adoptive cell therapy and ICB treatment [121]. Consequently, PD-L1 expression regulators identified via genetic screening represent potential pharmacological targets for ICB combination therapies [84, 89, 93, 127–129]. In a comprehensive genomic CRISPR/Cas9 screening within gastric cancer cells, identifying PD-L1^pos^ and PD-L1^neg^ sgRNA readouts via FACS revealed that Tripartite motif-containing 28 (TRIM28) is a crucial activator of PD-L1. Combining TANK Binding Kinase 1 inhibitors with anti-CTLA-4 demonstrated significant therapeutic efficacy in gastric cancer [128]. However, research suggests that increased PD-L1 expression in tumors may correlate with improved responses to anti-PD-1/PD-L1 therapies when combined with ICB [130]. Therefore, inhibiting the identified negative regulator of PD-L1, SET domain bifurcated histone lysine methyltransferase 1 (SETDB1), during ICB treatment has been found to simultaneously upregulate PD-L1 and enhance T-cell infiltration, resulting in a dual therapeutic effect [131].

ICB is a T-cell-based immunotherapy, and the factors affecting its efficacy are complex. Thus, the positive and negative regulatory factors found in in vitro and in vivo co-cultures of T cells and tumor cells can be modulated using engineered cells or small molecule inhibitors, with subsequent ICB application to validate efficacy and indirectly identify targets to enhance ICB therapy [104, 132–134]. Whole-genome CRISPR screening has revealed that Protein Arginine Methyltransferase 1 and Receptor Interacting Serine/Threonine Kinase 1 regulate cytotoxicity. Inhibiting these regulators can significantly boost the efficacy of anti-PD-1/TNF Receptor Superfamily Member 4 therapy in mice [132]. Consequently, the combined use of adoptive T cell therapy and ICB is a promising combination strategy, with gene screening directly or indirectly paving the way for these two types of immunotherapy.

### Bispecific T-cell engagers therapy

Bispecific T-cell engagers (BiTEs) have emerged as a significant immunotherapy strategy for cancer treatment in recent years. This approach involves concurrently binding the CD3 subunit of the TCR complex on cytotoxic T cells and TAAs on tumor cells. By redirecting T cells to target tumor cells, BiTEs increase recruitment and immune infiltration, leading to notable clinical outcomes. Resistance mechanisms to BiTE therapy are similar to those observed in adoptive T cell therapies, particularly CAR-T cells, and primarily involve the loss of tumor target antigens and other intrinsic tumor mechanisms [135]. High-throughput genetic screening, which involves transferring an sgRNA library into tumor cells and co-culturing them with CD8^+^ T cells and BiTEs, helps identify resistance mechanisms and targets in BiTE therapy. This approach provides rationales for patient selection and combination strategies with adoptive T cell therapies. Blinatumomab, an anti-CD19 BiTE, is the only clinically approved BiTE for treating B-ALL. Research by Li et al. revealed that loss of CD58, mediated by mutations in Paired Box 5, negates T cell activation induced by blinatumomab, highlighting its epigenetic mechanism of resistance[136].

In addition to anti-CD19 BiTEs, other BiTEs targeting various antigens in both hematological and solid tumors have been developed. The genetic resistance mechanisms of these BiTEs can be explored through high-throughput genetic screening [137–140]. For example, Flotetuzumab has shown therapeutic promise in relapsed/refractory AML. Whole-genome CRISPR screening identified a deficit in IFN-γ signaling as a key factor in tumor resistance [138]. Similarly, in vitro CRISPR KO and CRISPRa screening on tumor cells treated with Mesothelin and CD70 BiTEs identified the absence of factors such as Caspase-8, FADD-like apoptosis regulator, BCL2 Like 1, and BH3 interacting domain death agonist as key modulators of BiTE treatment sensitivity [140].

### Modulating different immune cell types in TME

**Fig. 4:**
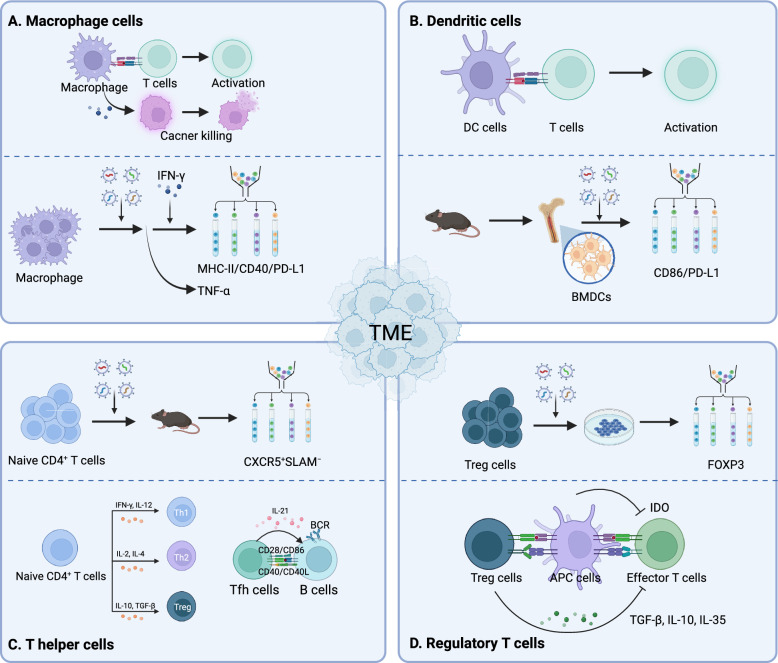
Genetic screening for modulating the TME. **A** Macrophages can either activate T cell functions or directly exert cytotoxic effects on tumors. Screening for macrophages involves isolating cells based on MHC-II, CD40, and PD-L1 expression under IFN-γ stimulation to evaluate their antigen-presenting capabilities, or assessing TNF-α levels to determine their cytotoxic potential. **B** DCs are the most important antigen-presenting cells, activating T cell functions. BMDCs are extracted from mice, and screening for CD86 and PD-L1 demonstrates their antigen-presenting capability. **C** CD4^+^ cells can differentiate into TH1, TH2, and Treg cells under the influence of various cytokines. CD4^+^ Tfh cells primarily aid in B cell proliferation and antibody production. In vivo screening of naïve CD4^+^ cells can identify Tfh cells through CXCR5^+^SLAM^−^. **D** Treg cells negatively impact T cell cytotoxicity, primarily through mediators such as TGF-β, IL-10, and IL-35. Regulators of Treg cells can be identified through screening for Foxp3

The TME plays a crucial role in tumor growth, metastasis, and prognosis, featuring immune cells with diverse functions that exert dual effects in adoptive T cell therapy. On one hand, the TME includes immune-activating cells like APCs and T follicular helper cells (Tfh), which can trigger potent immune cytotoxicity beneficial for tumor treatment. On the other hand, inhibitory cells in the TME, such as regulatory T cells (Tregs) and their secreted soluble factors, create an immunosuppressive environment that impedes T cell efficacy [141]. High-throughput genetic screening to identify targets in macrophages, dendritic cells, CD4^+^ T cells, and Treg cells within the TME can indirectly enhance the tumor-killing efficacy of adoptive T cell therapy (Fig. 4).

#### Immune-enhancing cells

M1-type macrophages are crucial in shaping and maintaining the TME. They exhibit innate immunity and the capability to phagocytize tumor cells, contributing to direct tumor elimination, and serve as APCs to activate cytotoxic T cells, thereby greatly advancing adoptive T cell therapy. Enhancing macrophage innate immunity through co-culturing with tumor cells [142, 143] and large-scale genetic editing screening, utilizing TNF-α to validate innate immune functions [144], is critical. Following IFN-γ stimulation, the assessment of antigen-presenting capacity via MHC-II, CD40, and PD-L1 promotes T cell activation [145, 146], offering novel targets for enhancing tumor immunity. For instance, glycogen synthase kinase 3β and mediator complex subunit 16, identified through screening, can regulate MHC-II expression influenced by IFN-γ [146].

Additionally, dendritic cells (DCs) are pivotal APCs within the body, central to initiating, regulating, and maintaining immune responses. Through whole-genome screening of bone marrow-derived DCs (BMDCs), the functional impact of CD86 and its regulators was uncovered. Co-culturing these genetically modified DCs with OT-I CD8 T cells, both in vitro and in vivo, confirms their antigen-presenting capacity, leading to T cell activation and cytotoxicity [147].

CD4^+^ helper T cells (Th), including Th1, Th2, Th17, and Tregs, exhibit unique functions and molecular features. Cytokine-mediated signaling often dictates their differentiation. High-throughput screening using FACS to sort cytokines, differentiation-related transcription factors, and specific surface receptors in activated CD4^+^ T cells helps to decipher key regulators of cell fate, a technique effectively utilized in Th1 and Th2 cells [148, 149]. Specifically, CD4^+^ Tfh cells, chiefly located in germinal centers and instrumental in their formation and upkeep, play a critical role in assisting B cell proliferation and antibody production, thereby contributing to humoral immunity. This understanding offers a fresh perspective in optimizing adoptive T cell therapy strategies [150]. By transferring CD4^+^ cells into mice bearing inflammation or tumors and analyzing sgRNA expression differences between Tfh (CXCR5^+^SLAM^−^) and Th1 (CXCR5^−^SLAM^+^) cells, researchers can identify the regulatory factors that influence Tfh differentiation [151, 152]. As an illustration, Huang and colleagues identified the inhibitory role of hypoxia-inducible factor-1 in Tfh, establishing a foundation for further modulation of the Th cell lineage within tumor immunology [152].

#### Immunosuppressive cells

Tregs, a distinct subset of CD4^+^ T cells, play a key role in modulating the body's immune response negatively through cell–cell interactions and the secretion of inhibitory cytokines. In the TME, Tregs often impede the effectiveness of adoptive T cell therapy, thereby contributing to tumor immune escape [153]. The central transcription factor Forkhead box P3 (Foxp3) is crucial for driving the Treg phenotype and maintaining Treg cell differentiation. Notably, Tregs that lack Foxp3 expression can regain the ability to produce pro-inflammatory cytokines, demonstrating the plasticity of Treg-mediated immune suppression [154]. High-throughput genetic screening, which involves introducing a comprehensive gene editing library into polarized Treg cell lines, has been instrumental in identifying regulatory targets and mechanisms affecting Foxp3 expression. By using FACS to sort Foxp3^high^ and Foxp3^low^ Treg populations and performing deep sequencing, researchers have uncovered both positive and negative regulators of Tregs [155–158]. For instance, Pinioti et al. discovered through CRISPR KO screening that the GDP-fucose transporter Slc35c1, along with broader fucose-related factors, positively regulates Foxp3. Knocking out these factors improved the immune functions of both CD4^+^ and CD8^+^ cells and significantly reduced colorectal cancer growth [157]. Similarly, ubiquitin-specific peptidase 22 (Usp22) has been shown to regulate Foxp3 expression at the transcriptional level, with Usp22 knockout demonstrating beneficial outcomes in several mouse tumor models [155]. In vivo screenings involving gene-edited MHC-II CD4^+^ cell subsets transplanted into mice with inflammation or tumors have further elucidated genes linked to immune responses. Validation studies revealed that methylenetetrahydrofolate dehydrogenase-2 deficiency could promote Treg cell differentiation, highlighting its potential as a target in tumor immunotherapy [159]. Additionally, mammalian target of rapamycin complex 1 (mTORC1) signaling is crucial for nutrient-driven cell metabolism, which impacts immune functions and plays a vital role in Tregs [160]. Long et al. performed α-CD3 continuous stimulation in nutrient-rich Treg polarized cell populations, analyzing sequencing data for mTORC1-dependent phosphorylation of S6 levels. Their protein–protein interaction network analysis identified positive and negative regulators of mTORC1 signaling. For instance, the Coiled-Coil Domain Containing 101-associated coactivator complex can restrict nutrient transport, and its specific depletion might enhance tumor immunity [161].

## Non-genetic screening applications for adoptive T cell therapies

By utilizing genetic screening, researchers can identify malfunctioning molecules within the metabolic pathways of tumors and cancers and apply existing technologies to target these molecules, thereby inhibiting or destroying tumors. Conversely, non-genetic screening can help clarify the genetic composition of drug-sensitive tumors, facilitating the development of targeted therapies or specific drugs. Additionally, it can identify the most suitable delivery vehicles to maximize drug transport to target sites. Integrating both approaches allows researchers to address dysfunctional molecules in the tumor signaling pathway by screening for appropriate therapeutic agents and combining drug therapy with cell therapy to enhance tumor-killing efficacy. Alternatively, researchers can identify abnormal molecular targets through genetic screening and subsequently find delivery vehicles with the highest affinity and specificity for delivering the corresponding drugs and cells, thereby improving the effectiveness of tumor treatment.

### Small-molecule compound screening

Small-molecule drugs have the potential to enhance adoptive T cell therapy by unexpectedly modulating immune pathways. Large-scale drug sensitivity screenings using co-cultures of tumor cells and T cells can identify optimal candidate drugs for combination tumor immunotherapy. When combined with genetic screening, this approach clarifies the mechanisms of action of small-molecule drugs, leading to improvements in the efficacy and safety of combination therapies. For instance, CD19^+^ CAR-T cells and NALM6 cell lines were co-cultured in multi-well plates with 526 different drugs, including chemotherapy agents, kinase inhibitors, epigenetic modulators, and non-cancer drugs. Evaluating cell viability revealed how these drugs affected CAR-T cytotoxicity. SMAC mimetics or inhibitors of apoptosis proteins antagonists, such as birinapant, Xevinapant, and LCL-161, were found to significantly enhance anti-tumor efficacy. Integrating this with CRISPR screening uncovered the sensitization mechanisms of SMAC mimetics and their association with death receptor expression [162]. Similarly, erlotinib, an EGFR inhibitor, emerged as a preferred compound for boosting CD8^+^ T cell-mediated cytotoxicity in ovarian cancer cells [163].

Immune and cancer cells share numerous essential metabolic pathways, leading to intense nutrient competition within the TME. Metabolic reprogramming in both tumor cells and T cells plays a crucial role in tumor immunity, particularly in pancreatic ductal adenocarcinoma. Genetic screening is employed to explore fundamental metabolic pathways during pancreatic tumor progression and TME-induced dependencies, identifying potential therapeutic targets [164, 165]. To pinpoint the metabolic pathways critical for T cell activation, a screening of a metabolite inhibitor library was conducted on sgRNA-modified Jurkat T cells and primary human T cells. By tracking T cell activation markers (CD69, ICOS7, and CD25), nicotinamide phosphoribosyltransferase (NAMPT), an essential enzyme in NAD^+^ biosynthesis, was identified as crucial for T cell activation. This suggests that NAD^+^ supplements could enhance T cell-based immunotherapy [47].

Currently, small-molecule drug screening faces challenges, including uncertainty about the specific mechanisms of action of the screened drugs and their effects being limited to a small number of genes. Moreover, translating these findings into clinical applications requires careful consideration of drug toxicity and potential impacts on normal bodily functions, which may worsen the condition or cause severe adverse reactions. Addressing these challenges is essential. In the future, integrating drug screening with immunotherapy and gene therapy may offer a multi-faceted approach to targeting tumors, thereby inflicting damage and enhancing therapeutic efficacy.

### Targeted delivery system screening

**Fig. 5:**
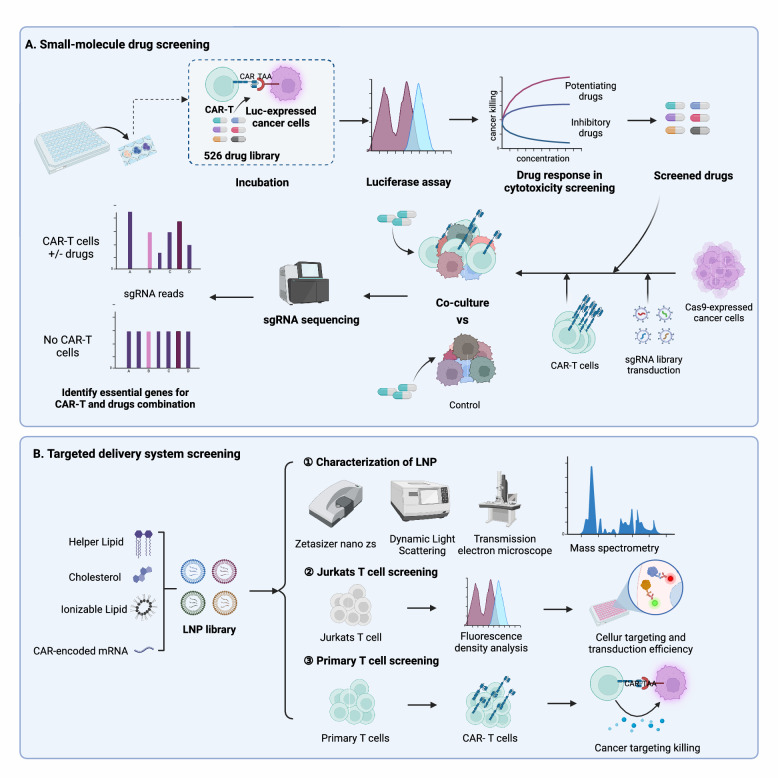
Non-genetic screening applications for adoptive T cell therapies. **A** To identify the optimal combination of drugs that enhance the tumoricidal effect of CAR-T cells, a drug library is added to tumor cells and CAR-T cells within 96-well or 384-well plates. The tumor-killing effect is screened using Luc assays to identify drugs that either potentiate or inhibit the efficacy of CAR-T therapy. The identified drugs are then co-cultured with tumor cells engineered with an sgRNA library, with or without concurrent CAR-T treatment. This co-culture helps to uncover the molecular mechanisms underlying any observed drug synergy through combined genetic screening. **B** For transient CAR-T cells using mRNA, LNPs consist mainly of four components: auxiliary lipid, cholesterol, ionizable lipid, and mRNA. Orthogonal library design is used to optimize the first three components. Transduction efficiency and cell targeting of LNPs are assessed through material characterization and Jurkat T cell line experiments, with the CAR-T cells constructed in primary T cells to verify their tumor-killing function

TCR-T and CAR-T cells utilize lentiviral or retroviral vectors to randomly integrate plasmids encoding the TCR α and β chain genes from effector T cells, which are induced by tumor antigens, or scFvs that specifically recognize tumor antigens, into the genome of peripheral blood T cells. This integration ensures stable, continuous expression through cell division. However, this method carries risks, such as potential tumor formation due to insertional mutagenesis. Additionally, the complexity of producing clinical-grade viral vectors leads to high costs and extended production times, which can reduce clinical accessibility and increase the risk of T cell exhaustion following prolonged activation [166]. As a result, mRNA-mediated adoptive cell infusion therapies have emerged as a promising alternative. These therapies benefit from non-integrative expression and simplified non-viral delivery systems, which enhance control over therapeutic pharmacokinetics. This method improves the efficacy, safety, and accessibility of adoptive T cell therapies [167]. In this approach, transiently expressed CAR-T cells are generated by introducing mRNA encoding the CAR sequence into autologous CD8^+^ T cells ex vivo, using techniques such as electroporation or LNP delivery. The anti-tumor efficacy and safety of this method have been demonstrated in various in vitro and in vivo tumor models [168–172]. Similarly, introducing mRNA for TCR sequences into T cells ex vivo to create TCR-T cells has been shown to induce strong anti-tumor immunity [170] (Fig. 5A).

LNPs are commonly used for mRNA delivery but often accumulate in the liver, lacking specificity for T cells. High-throughput screening of various LNP formulations for mRNA delivery in Jurkat cell lines is employed to identify those with enhanced affinity and efficacy for T cells. This screening involves assessing protein expression, cell function, and tumor cell co-culture [173–175], thereby laying the groundwork for non-viral adoptive T cell therapies. For instance, Billingsley et al. conducted high-throughput screening in Jurkat cell lines and primary human T cells, identifying C14-4 as the most effective ionizable lipid for LNPs in T cells. They validated its effectiveness in CAR-T cell engineering by expressing the CD19 CAR gene and demonstrated its efficacy in targeting ALL tumor cells [175] (Fig. 5B).

In carrier screening, specificity remains a critical limitation. Researchers must focus on identifying the most suitable components for targeting specific organs through screening. Additionally, screening the structures of delivered mRNA is essential for comparing different mRNAs, as well as their effects on stability and expression efficiency. This enhances targeting design and helps identify optimization patterns. Furthermore, screening improves the controllability of targeted delivery and allows a more comprehensive evaluation of safety, toxicity, and stability in clinical applications. The stability of the carrier is vital for enhancing delivery efficiency, as stable carriers can transport the maximum amount of drug to target organs and cells. Future efforts should leverage carrier screening to select suitable carriers, enabling effective drug delivery to various target cells and maximizing therapeutic efficacy [176].

## Conclusion and future perspective

Adoptive T cell therapy represents a significant advancement in cancer immunotherapy research. Emerging high-throughput screening technologies provide an unbiased means to uncover resistance mechanisms and propose optimization strategies. This article focuses on CRISPR screening within high-throughput gene screening as an advanced tool, highlighting its superior flexibility and editing efficiency compared to siRNA and shRNA methods. CRISPR-based screening enhances accuracy and safety, establishing it as a leading method in current gene screening practices. The article also emphasizes specific applications of gene screening technologies in optimizing adoptive cell therapy, including T cell screening, tumor cell screening under T cell pressure, and various combination strategy screenings. The advancement of high-throughput technologies reflects the trend towards precision and specificity in molecular biology, especially in light of detailed research. The rapid development of biotechnologies and their complementary advantages set the stage for enhancing T cell-related therapies, including adoptive cell therapy and ICB therapies.

Despite the potential of gene-editing technologies like siRNA, shRNA, and CRISPR for cancer therapy, several limitations and challenges hinder their widespread clinical translation. A primary concern is off-target effects, leading to unintended gene silencing, which impacts both therapeutic efficacy and safety. Efficient delivery, especially for systemic administration, remains a significant obstacle, including challenges such as cellular uptake, endosomal escape, and targeted delivery to tumor cells. The high cost of development and manufacturing, particularly for personalized CRISPR therapies, poses a significant barrier, especially when considering large-scale clinical implementation and the complexities of delivering these gene-editing tools. Additionally, tumor heterogeneity and the potential for acquired resistance underscore the need for combinatorial therapies and personalized treatment strategies. Future research should prioritize enhancing target specificity, developing more efficient and biocompatible delivery systems, and reducing manufacturing costs. Addressing these challenges is crucial for translating promising preclinical findings into effective and accessible cancer therapies, ultimately revolutionizing cancer treatment. Developing robust preclinical models that accurately recapitulate human tumor biology and enable rigorous evaluation of efficacy and safety prior to clinical translation is essential. This advancement will support the development of next-generation RNAi and CRISPR-based therapies capable of targeting a broad spectrum of cancer types and achieving durable clinical responses.

The establishment and transduction of large-scale screening libraries enable researchers to identify new targets and uncover the mechanisms of interaction between tumor cells and adoptive T cell therapy, with validation through computational and experimental approaches. Nonetheless, enhancing the sensitivity and efficiency of high-throughput screening technologies in tumor immunology research is a critical challenge [177]. Firstly, the use of CRISPR variants has consistently improved precision, efficiency, and safety, as well as expanded the scope of target identification. However, it also faces challenges related to unpredictable off-target risks [178]. High-throughput sequencing technologies can be used for sensitive and efficient detection of off-target effects. Integrating these technologies with machine learning models to predict gene editing efficiency and off-target probabilities, and capitalizing on large-scale sample analysis, can provide more objective insights into screening outcomes [179]. Secondly, high-throughput data generation exceeds traditional experimental methods in speed and volume. The standardization and reproducibility of these high-throughput techniques are vital for data reliability. Technical discussions are needed to maintain sample purity and minimize irrelevant factor interference while increasing screening throughput [180]. With the gradual commercialization of microfluidics and droplet sorting technologies, we anticipate the development of more sensitive high-throughput systems, subject to stricter standardized operating procedures and quality control measures [181]. High-throughput screening technologies, with their precision and specificity, can enhance the controllability of T cell therapies [182]. They offer comprehensive means to assess safety, toxicity, and stability in clinical applications. Combined with other therapeutic approaches, these technologies facilitate synergistic effects, enhancing the likelihood of clinical translation.

Effective delivery of CRISPR screening components into cell pools is essential for successful screening. Delivery systems primarily involve viral vectors and electroporation [183]. Non-viral carriers such as liposomes, polymers, and LNPs are increasingly used for their simplicity, scalability, and controllable targeting. These non-viral systems, with their larger packaging capacity and reduced immunogenicity, offer significant potential, particularly in improving target specificity in high-throughput screening and facilitating in vivo applications [184]. CRISPR screening primarily involves sgRNA and Cas9 protein. Beyond delivery methods, optimizing these components can significantly improve their functional efficiency [185]. For instance, stabilizing sgRNA signals and extending their detection without signal decay present challenges. Utilizing high-fidelity engineered nucleases, such as Cas-CLOVER, HF1, and HiFi (High-precision, long-read genome sequencing technology), enhances DNA cutting accuracy, thus minimizing off-target effects associated with Cas proteins [186].

In summary, this review explores the potential of advanced high-throughput screening in adoptive T cell therapies and combination strategies for malignant tumors. By leveraging continuous improvements in high-throughput screening technologies and tumor immunotherapy, targets and downstream pathways can be optimized through engineering techniques or small molecule medications. This progress is poised to energize the future landscape, potentially revolutionizing disease diagnosis, treatment, and personalized medicine.

## Data Availability

No datasets were generated or analysed during the current study.

## Abbreviations

AAV: Adeno-associated virus
ADAM2: ADAM Metallopeptidase Domain 2
ALL: Acute lymphoeytic leukemia
AML: Acute myeloid leukemia
AP4: Transcription factor
APCs: Antigen-presenting cells
Arid1a: AT-Rich Interaction Domain 1A
ASF1A: Anti-Silencing Function 1A Histone Chaperone
ATAC-seq: Assay for Targeting Accessible-Chromatin with high-throughput sequencing
B-ALL: B cell-acute lymphoblastic leukemia
BAF: B cell-activating factor
BATF: Basic Leucine Zipper ATF-Like Transcription Factor
BCL: B cell lymphoma
BCL2L1: BCL2 Like 1
BCMA: B cell maturation antigen
BCP-ALL: B cell precursor acute lymphoblastic leukemia
BE: Base editor
BHLHE40: Basic Helix-Loop-Helix Family Member E40
BID: BH3 interacting-domain death agonist
BiTE: Bispecific T-cell engagers
BMDCs: Bone marrow-derived DCs
BTN2A1: Butyrophilin Subfamily 2 Member A1
BTN3A: Butyrophilin Subfamily 3 Member A
CAF-1: Chromatin assembly factor
CAR: Chimeric antigen receptor
Cas12a: CRISPR-associated protein 12a
Cas9: CRISPR-associated protein 9
CASTOR1: Cytosolic Arginine Sensor For MTORC1 Subunit 1
cBAF: Canonical Brgl/Brg-associated factor
CCC: Combinatorial Cellular Library
CCDC101: Coiled-Coil Domain Containing 101
CCNC: Cyclin C
CCR7: C–C motif chemokine receptor
CD: Cluster of differentiation
CDK: Cycle-dependent kinase
CDRH3: Complementary determining region
CFLAR: Caspase-8, FADD-like apoptosis regulator
CFSE: Carboxyfluorescein succinimidyl amino ester
c-FLIP: Cellular FLICE-inhibitory protein
CLASH: CRISPR-based library-scale AAV perturbation with simultaneous HDR knock-in
CMTM6: CKLF Like MARVEL Transmembrane Domain Containing 6
CRISPR: Clustered Regularly Interspaced Short Palindromic Repeats
CRISPR KI: CRISPR Knock in
CRISPR KO: CRISPR Knock out
CRISPRa: CRISPR action
CRISPRi: CRISPR interfrence
crRNA: CRISPR RNA
CTL: Cytotoxic T cells
CTLA-4: Cytotoxic T-Lymphocyte-Associated protein 4
DC: Dendritic cell
dCas9: Deactivated Cas9
DHX37: DEAH-Box Helicase 37
DISC: Death-inducing signaling complex
DLS: Dynamic light scattering
DNTs: Double-negative T cells
DSB: DNA double-strand breaks
EBF4: Early B cell factor 4
EGFR: Epidermal Growth Factor Receptor
EMT: Epithelial-mesenchymal transition
Epi: Epithelial
ETS1: ETS Proto-Oncogene 1
FACS: Fluorescence-activated cell sorting
FAM49B: Family with sequence similarity 49 member B
FASL: Factor-related Apoptosis ligand
Foxp3: Forkhead box P3
Fuco: Fucosylation
Fut8: Fucosyltransferase 8
GCs: Germinal centers
GM-CSF: Granulocyte–macrophage colony-stimulating factor
GOF: Gain-of-function
GPR183: G Protein-Coupled Receptor 183
GSEA: Gene set enrichment analysis
GSK3β: Glycogen Synthase Kinase 3 β
HDR: Homology-directed repair
HF1: H Factor 1
HIF-1α: Hypoxia-inducible factor-1
HiFi: High-precision, long-read genome sequencing technology
IAP: Inhibitors of apoptosis proteins
ICAM-1: Intercellular adhesion molecule 1
ICB: Immune checkpoint blocks
ICDs: Intracellular domains
IFN-γ: Interferon-γ
IKZF2: Ikaros family zinc finger protein 2
IL10RB-DT: IL10RB Divergent Transcript
INO80: INO80 Complex ATPase Subunit
IRS: Immune response suppressors
ITAM: Immunoreceptor tyrosine-based activation motif
JAK: Janus Kinase
KRAB: Kruppel-associated box
LFA-1: Lymphocyte function-associated antigen 1
lncRNA: Long non-coding RNA
LNP: Lipid nanoparticles
LOF: Loss-of-function
LTBR: Lymphotoxin-β receptor
LUBAC: Linear ubiquitin chain assembly complex
Luc: Luciferase
MED16: Mediator complex subunit 16
Mes: Mesenchymal-like
Mfge8: Milk Fat Globule-EGF Factor
MHC: Major histocompatibility complex
MHC-I APP: MHC-I antigen processing pathway
MM: Multiple myeloma
ModPoKI: Modular Pooled Knockin
MP: Memory precursor cells
mRNA: Messenger RNA
MSLN: Mesothelin
mSWI/SNF: Mammalian SWItch/Sucrose Non-Fermentable
MTHFD2: Methylenetetrahydrofolate dehydrogenase-2
mTORC1: Mechanistic target of rapamycin complex 1
NAMPT: Nicotinamide phosphoribosyltransferase
nCas9: Nickase Cas9
NFATC2: Nuclear Factor of Activated T Cells 2
NF-κb: Nuclear factor kappa-B
NK: Natural killer
NR4A1/3: Nuclear receptor subfamily 4 group A member 1/3
NUDT21: Nudix Hydrolase 21
ORF: Open reading frame
OTOT: On-target, off-tumor
OX40: TNF Receptor Superfamily Member 4
P38: A class of mitogen-activated protein kinases
PAX5: Paired Box 5
PCR: Polymerase chain reaction
PDAC: Pancreatic ductal adenocarcinoma
PD-1: Programmed cell death 1
PD-L1: Programmed Death Ligand 1
PIK3CD: Phosphoinositide-3-kinase catalytic subunit delta
PLCγ-1: Phospholipase C γ 1
PPI: Protein–protein interaction
PRDM1: PR/SET Domain 1
PRMT1: Protein Arginine Methyltransferase 1
PRODH2: Proline Dehydrogenase 2
p-S6: Phosphorylation of S6
RASA: RAS P21 Protein Activator
RIPK1: Receptor Interacting Serine/Threonine Kinase 1
RNAi: RNA interference
S1PR1: Sphingosine-1-phosphate receptor
SAGA: Spt-Ada-Gcn5-acetyltransferase
SATB1: Special AT-Rich Sequence Binding Protein 1
scCAR-seq: Single-cell CAR sequencing
scCRISPR: Single-cell CRISPR
scFv: Single-chain variable fragment
scRNA-seq: Single-cell RNA sequencing
SET: SET Nuclear Proto-Oncogene
SETDB1: SET domain bifurcated histone lysine methyltransferase 1
sgRNA: Single guide RNA
Slc35c1: Solute Carrier Family 35 Member C1
SNX9: Sorting nexin 9
St3gal1: ST3 β-galactoside α-2,3-sialyltransferase 1
STAT1: Signal Transducer and Activator of Transcription 1
STUB1: STIP1 Homology And U-Box Containing Protein 1
TAA: Tumor-associated antigens
TCR: T cell receptor
TE: Term effector cells
T_EFF_: T effector cell
TFAP4: Transcription Factor AP-4
Tfh: T follicular helper cells
Th: CD4^+^ helper T cells
TILs: Tumor-infiltrating lymphocytes
TLE4: Transducin-like enhancer of split 4
TM: Transmembrane domain
TME: Tumor micro-environment
T_MEM_: T memory cell
TNF: Tumor necrosis factor
TRAC: T Cell Receptor Alpha Constant
TRAIL: TNF-related apoptosis-inducing ligand
Treg: Regulatory T cell
TRIM28: Tripartite motif-containing 28
TSA: Tumor-specific antigens
TSS: Transcription start site
Usp22: Ubiquitin Specific Peptidase 22
VP64: Virus protein 64
